# In vivo transcriptional analysis of mice infected with *Leishmania major* unveils cellular heterogeneity and altered transcriptomic profiling at single-cell resolution

**DOI:** 10.1371/journal.pntd.0010518

**Published:** 2022-07-05

**Authors:** Gopinath Venugopal, Jordan T. Bird, Charity L. Washam, Hayden Roys, Anne Bowlin, Stephanie D. Byrum, Tiffany Weinkopff

**Affiliations:** 1 Department of Microbiology and Immunology, College of Medicine, University of Arkansas for Medical Sciences, Little Rock, Arkansas, United States of America; 2 Department of Biochemistry and Molecular Biology, College of Medicine, University of Arkansas for Medical Sciences, Little Rock, Arkansas, United States of America; 3 Arkansas Children’s Research Institute, Little Rock, Arkansas, United States of America; Centro de Pesquisa Gonçalo Moniz-FIOCRUZ/BA, BRAZIL

## Abstract

*Leishmania* parasites cause cutaneous leishmaniasis (CL), a disease characterized by disfiguring, ulcerative skin lesions. Both parasite and host gene expression following infection with various *Leishmania* species has been investigated in vitro, but global transcriptional analysis following *L*. *major* infection in vivo is lacking. Thus, we conducted a comprehensive transcriptomic profiling study combining bulk RNA sequencing (RNA-Seq) and single-cell RNA sequencing (scRNA-Seq) to identify global changes in gene expression in vivo following *L*. *major* infection. Bulk RNA-Seq analysis revealed that host immune response pathways like the antigen processing and presentation pathway were significantly enriched amongst differentially expressed genes (DEGs) upon infection, while ribosomal pathways were significantly downregulated in infected mice compared to naive controls. scRNA-Seq analyses revealed cellular heterogeneity including distinct resident and recruited cell types in the skin following murine *L*. *major* infection. Within the individual immune cell types, several DEGs indicative of many interferon induced GTPases and antigen presentation molecules were significantly enhanced in the infected ears including macrophages, resident macrophages, and inflammatory monocytes. Ingenuity Pathway Analysis of scRNA-Seq data indicated the antigen presentation pathway was increased with infection, while EIF2 signaling is the top downregulated pathway followed by eIF4/p70S6k and mTOR signaling in multiple cell types including macrophages, blood and lymphatic endothelial cells. Altogether, this transcriptomic profile highlights known recruitment of myeloid cells to lesions and recognizes a potential role for EIF2 signaling in murine *L*. *major* infection in vivo.

## Author summary

*Leishmania major* parasites cause cutaneous leishmaniasis, which is characterized by disfiguring, ulcerative skin lesions. Here, we show murine *L*. *major*-directed reprogramming of the host transcriptome in vivo. Our bulk RNA-Seq analyses revealed upregulation of antigen processing and presentation pathway, while the host ribosomal pathway was downregulated following *L*. *major* infection. Similarly, scRNA-Seq analyses revealed the upregulation of transcripts responsible for antigen presentation and host defense proteins like guanylate binding proteins (GBPs) alongside the downregulation of EIF2 signalling at the site of *L*. *major* infection. Overall, our transcriptomic dataset provides a comprehensive list of gene expression at the single-cell resolution, and highlights a previously undefined role for EIF2 signalling during murine *L*. *major* infection in vivo.

## Introduction

Leishmaniasis is a multifaceted disease caused by different species of obligate intracellular protozoan parasites of the genus *Leishmania*, belonging to the Trypanosomatid family. Depending on the complex interaction between the species and the host immune system, the disease can vary in severity resulting in a wide spectrum of clinical outcomes that have been classified into the following categories: cutaneous leishmaniasis (CL), mucocutaneous leishmaniasis (MCL), disseminated leishmaniasis or diffuse CL (DCL) where lesions remain localized to skin or mucosal surfaces, and a life-threatening condition called visceral leishmaniasis (VL) where parasites migrate to the internal organs like the liver, spleen and bone marrow [1]. As per the World Health Organization (WHO), leishmaniasis is a major public health problem due to its annual incidence of 1.7 million new cases worldwide [1,2].

Of the species belonging to subgenus *Leishmania*, *L*. *major* is an important etiological agent of CL and possesses clinical and epidemiological importance, especially in parts of Asia, the Middle East, Northern Africa, and Southern Europe [3]. Although CL is not fatal and considered to be a self-healing disease, the development of nodules or papules followed by ulcerations at the site of infection is the hallmark of the disease; importantly, both parasite replication and the host immune response can contribute to the disease pathology [3]. CL produces permanently disfiguring skin lesions that are associated with massive immune cell recruitment, across the blood vascular endothelium, and into the skin where the parasite resides [4–8]. In addition to parasite control, expansion of the lymphatics is required for lesion resolution [9,10]. While CD4^+^ Th1 cells producing IFNγ are required to activate macrophages to kill parasites via NO, the exacerbated activation and sustained recruitment of immune cells including neutrophils, NK cells, Ly6C^+^ inflammatory monocytes, and CD4^+^ and CD8^+^ T lymphocytes induces a chronic inflammatory response; this chronic inflammation leads to tissue necrosis and skin damage, a feature of non-healing lesions [11–13].

Myeloid cells such as monocytes and macrophages are the primary permissive host cells for *Leishmania* parasites, and elimination of parasites by these cells is critical for host resistance [11,14–16]. While the importance of macrophages as host cells for parasites and effector cells involved in parasite killing has been well-characterized, emerging evidence suggests Ly6C^+^CCR2^+^ inflammatory monocytes are a preferential target for parasites and serve as the initial myeloid host cell for early parasite replication; however, monocytes also perform effector functions by producing inducible nitric oxide (iNOS) in an IFNγ-dependent manner to kill parasites [14,16–19]. In addition to the parasite taking advantage of the Th1-mediated recruitment of inflammatory monocytes for infection, recruited neutrophils and monocyte-derived dendritic cells at the site of infection can also harbor parasites, suggesting these immune cells serve an important function in host-parasite interplay [16,20–26].

*Leishmania* parasites possess a variety of virulence mechanisms [27,28] and use several strategies to evade the host immune response for intracellular survival including modulating the host immune response by altering T cell responses, impeding antigen display by MHCII, and hindering nitric oxide (NO) production [29,30]. NO not only directly kills parasites, but NO also regulates the breadth of the inflammatory response through quorum sensing and restricts the supply of proliferation-permissive host cells including monocyte-derived phagocytes to the site of infection [26,31,32]. Importantly, *Leishmania* parasites can escape from oxidative burst and they fail to activate optimal macrophage innate immune responses [33,34]. Despite the robust Th1 response and NO production, parasite growth persists in CD206^+^ tissue-resident dermal macrophages in non-healing *L*. *major* infection suggesting cells in inflamed skin respond to soluble tissue mediators in the microenvironment differently. As a result, we set out to identify changes at the transcriptional level following *Leishmania* infection within different cell types and especially within the hostile tissue microenvironment.

Until recently, many studies examining the host response relied mostly on microarray-based or serial analysis of gene expression tag approaches. Using these approaches, previous studies compared the host gene expression profile between infection with promastigotes and amastigotes [35–39] or different species of *Leishmania* [40–42]. However, microarray-based approaches have technical limitations such as hybridization and cross hybridization artefacts, dye-based detection issues, certain probes cannot be included on the microarrays, and the inability to detect 5’ and 3’ UTRs boundaries [43,44]. In recent years, RNA-Sequencing (RNA-Seq) has emerged as a powerful tool to study transcriptional changes and also significantly enhanced our understanding of disease pathogenesis due to its high sensitivity. Moreover, transcriptomic profiling using RNA-Seq following infection with various species of *Leishmania* has been mostly applied to in vitro experiments and some studies have investigated transcriptional changes in the human or murine host as well as the parasite simultaneously [45–52]. A recent study comparing the gene expression profile of cutaneous lesions from *L*. *braziliensis*-infected patients with or without treatment revealed most of the differentially expressed transcripts were correlated with cytotoxicity-related pathways and parasite loads [49]. The same group also revealed a consistent myeloid interferon stimulated gene (ISG) signature in skin lesions from *L*. *braziliensis*-infected patients using RNA-Seq [53]. Altogether, these studies revealed that the host immune response upregulates transcripts related to both pro- and anti-inflammatory responses during leishmaniasis [54,55]. Although the key biological mechanisms involved in the pathogenesis of CL have been well characterized using both in vitro and in vivo models, many of the mechanistic studies in vivo were investigated in the context of a single cytokine or only examining one to few cell types at a time, which does not reflect the complex inflammatory environment of leishmanial lesions. Additionally, many studies predominantly focused on the immune cells and neglected stromal cells such as the endothelial cells, so we aimed to perform a study that encompasses all the cell types located in lesions that may participate in pathogen control and/or the pathogenesis of disease. Furthermore, none of the above RNA-Seq studies to date have characterized the global transcriptional reprogramming following *L*. *major* infection in vivo, which is the most widely used murine model of CL to study disease pathogenesis and parasite-host interactions. The *Leishmania* field also lacks a comprehensive murine transcriptomic profile that could identify novel transcriptomic changes and new pathways within individual cell types in vivo that could lead to exciting new avenues for therapeutic interventions. Therefore, our study combines both bulk RNA-Seq and scRNA-Seq to assemble a comprehensive dataset that defines how the host response reprograms individual cell types following *L*. *major* infection to determine the molecular mediators contributing to the pathogenesis of disease.

## Results

### Enriched pathways for differentially expressed genes during *L*. *major* infection revealed by bulk RNA-Seq

To characterize the transcriptomic landscape during *L*. *major* infection in vivo, we applied RNA-Seq analysis on ear samples obtained from mice that were infected with *L*. *major* promastigotes and uninfected naive control ears. In this experimental murine model of *L*. *major* infection, lesion volume peaks between 3–5 weeks post-infection (p.i.) (S1 Fig) and then the lesion starts to resolve spontaneously about 8 weeks p.i. after parasites have been controlled by a Th1 immune response; full lesion resolution typically occurs about 12 weeks p.i. For parasite burdens, at 4 weeks p.i, an average of 2–2.5x10^9^ parasites are present in lesions and parasite numbers decline over time as the lesion resolves. As a result, the host transcriptome was investigated from ear samples collected from experimentally-infected mice at 4 weeks p.i. and compared to naive controls [56].

**Fig 1:**
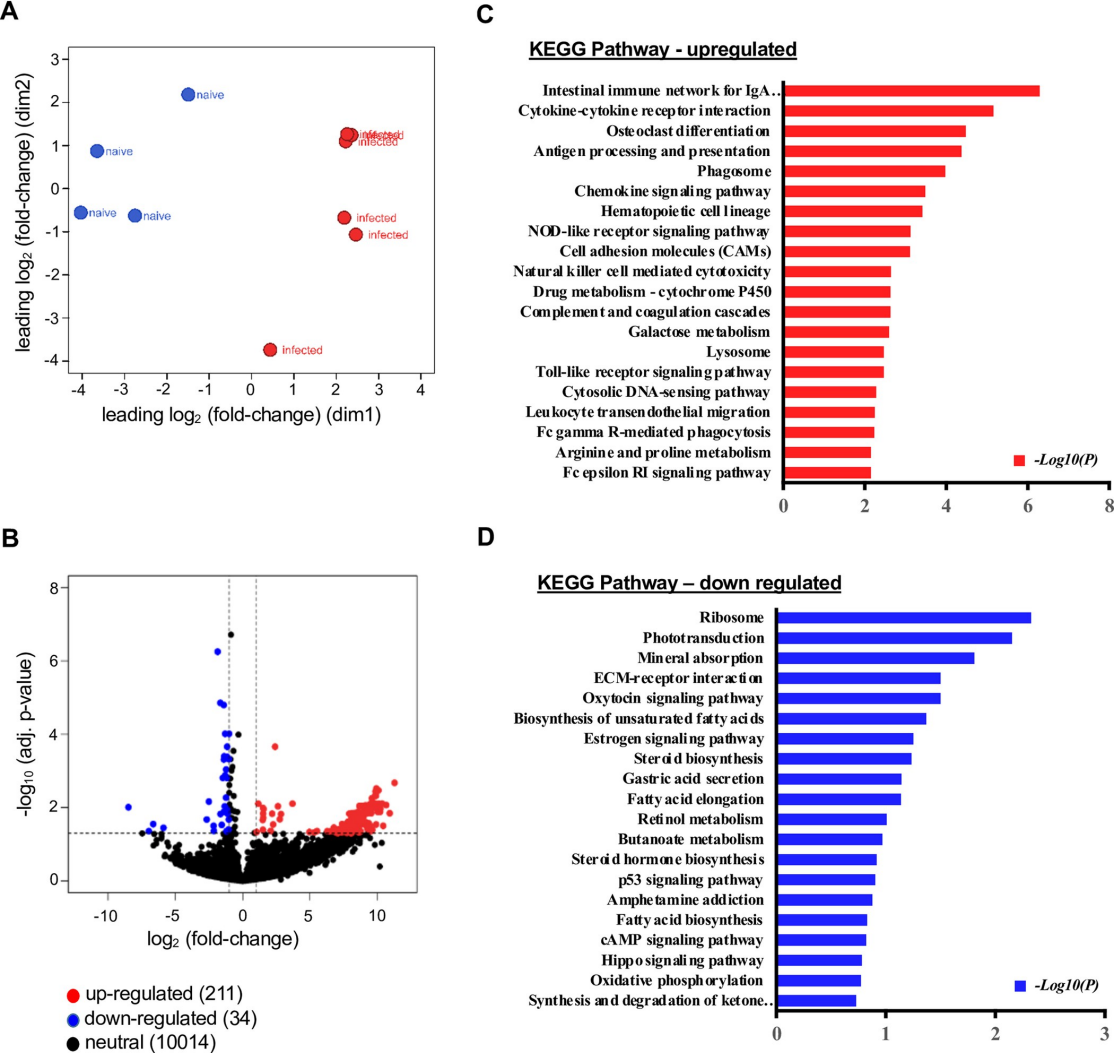
*Leishmania* infection is associated with differential regulation of host immune response pathways in vivo. **(A)** Multi-Dimensional Scaling (MDS) plot showing the gene expression profile between naive and *L*. *major-*infected ears. **(B)** Volcano plot of all DEGs in naive and infected ears. Red dots represent DEGs with increased RNA abundance using a log2FC >1 and p-value <0.05. Blue represents DEGs with decreased RNA abundance using a log2FC <−1 and p-value <0.05. Only annotated genes are shown in plot. FC, fold-change. **(C-D)** Bulk RNA-Seq analysis indicates DEGs that are highly correlated with signaling pathways. KEGG enrichment analysis of top 20 upregulated (C), and top 20 downregulated (D) pathways enriched among the DEGs between naive and *L*. *major*-infected ears (pathways selected by significant and FC > 1.5, list includes rank, significance and adjusted average). Data are shown from 4 naïve control and 6 *L*. *major* infected mice.

To compare the gene expression profiles of infected and naive mice, bulk RNA-Seq was performed on *L*. *major*-infected ears and naive control ears. Transcriptional analysis revealed that ears from infected mice and naive controls were distinct from one another, as determined by multidimensional scaling (MDS) plot and DEG analysis. MDS plot shows the positions of each sample, with samples from different experimental groups being well separated, and samples from the same experimental group clustering together (Fig 1A). Therefore, the distance between samples reveals the distinct pattern of gene expression between the infected and naive animals (Fig 1A). To investigate transcriptomic signatures associated with infection, we carried out DEG analysis between infected mice and naive controls by comparing the RNA-Seq read counts of the various genes and subsequently applying the cut-off criteria. Transcripts that were either increased or decreased in RNA abundance with infection (logCPM><1) were included in the volcano plot showing transcriptional differences observed between infected and naive ears (Fig 1B). The gene expression profiles derived from the RNA-Seq data were calculated using the RPKM method [57] and a fold change >2 and p<0.05 were considered statistically significant. Of more than 10,800 genes that were detectable in infected ears, we observed that 211 transcripts were increased in RNA abundance and 34 transcripts were decreased in RNA abundance with infection, while 10,014 genes did not show any significant differences between naive and *L*. *major*-infected ears (Fig 1B). Here, it should be noted that Gene Set Enrichment Analysis (GSEA) was carried out against the KEGG pathways and involved a ranked order list which contains both DEG and fold changes for more than 10800 genes. The complete list of genes detected in our RNA-Seq analysis is listed in S1 Table.

GSEA using KEGG pathways revealed a total of 276 enriched pathways which includes pathways involved in both disease conditions and molecular signaling networks. Specifically, the antigen processing and presentation pathway was found to be significantly enriched amongst DEGs, while the ribosomal pathway was significantly downregulated during *L*. *major* infection (Fig 1C and 1D). In addition to antigen processing and presentation, we observed many other host immune response pathways upregulated with infection including: cytokine-cytokine receptor interaction, phagosome, chemokine signaling, cell-adhesion molecules pathway, NK cell mediated cytotoxicity, leukocyte trans-endothelial migration and Fcγ receptor-mediated phagocytosis (Fig 1C). Conversely, top 20 downregulated pathways enriched for DEGs in *L*. *major* infection were related to ribosomal translation, mineral absorption, ECM-receptor interaction, biosynthesis of unsaturated fatty acids, and steroid biosynthesis (Fig 1D). The top 10 KEGG pathways for both upregulated and downregulated pathways with the fold change and adjusted ‘*p*’ value are listed in Table 1. A number of disease-specific KEGG pathways appeared prominent in the enrichment analysis including *Staphylococcus aureus* infection, autoimmune thyroid disease, and graft vs. host disease (S2 Table). Importantly, leishmaniasis emerged as one of the top disease pathways highlighting the quality of the input data for the analysis (S2 Table).

**Table 1:** Differentially expressed genes (DEGs) enriched for top 10 KEGG pathways following *L*. *major* infection.

Pathway regulation	KEGG enriched pathways	Avg. log-fold change	Adj. p value
**Enriched**	Intestinal immune network for IgA production	5.23228	5.08E-07
	Cytokine-cytokine receptor interaction	8.337469	6.97E-06
	Osteoclast differentiation	7.623207	3.31E-05
	Antigen processing and presentation	4.468822	4.21E-05
	Phagosome	6.527853	0.000106
	Chemokine signaling pathway	8.329182	0.000328
	Hematopoietic cell lineage	6.848572	0.000381
	NOD-like receptor signaling pathway	6.638004	0.000744
	Cell adhesion molecules	6.486765	0.000769
	Natural killer cell mediated cytotoxicity	8.022157	0.002282
**Depleted**	Ribosome	-0.696061	0.004705
	Phototransduction	-0.907101	0.007031
	Mineral absorption	-0.992836	0.015538
	ECM-receptor interaction	-0.889139	0.031673
	Oxytocin signaling pathway	-0.948893	0.031673
	Biosynthesis of unsaturated fatty acids	-1.474018	0.042932
	Estrogen signaling pathway	-1.199847	0.055815
	Steroid biosynthesis	-0.908215	0.058351
	Gastric acid secretion	-0.907101	0.072276
	Fatty acid elongation	-0.892548	0.072946

### Differential gene expression in immune-related pathways during *L*. *major* infection revealed by bulk RNA-Seq

**Fig 2:**
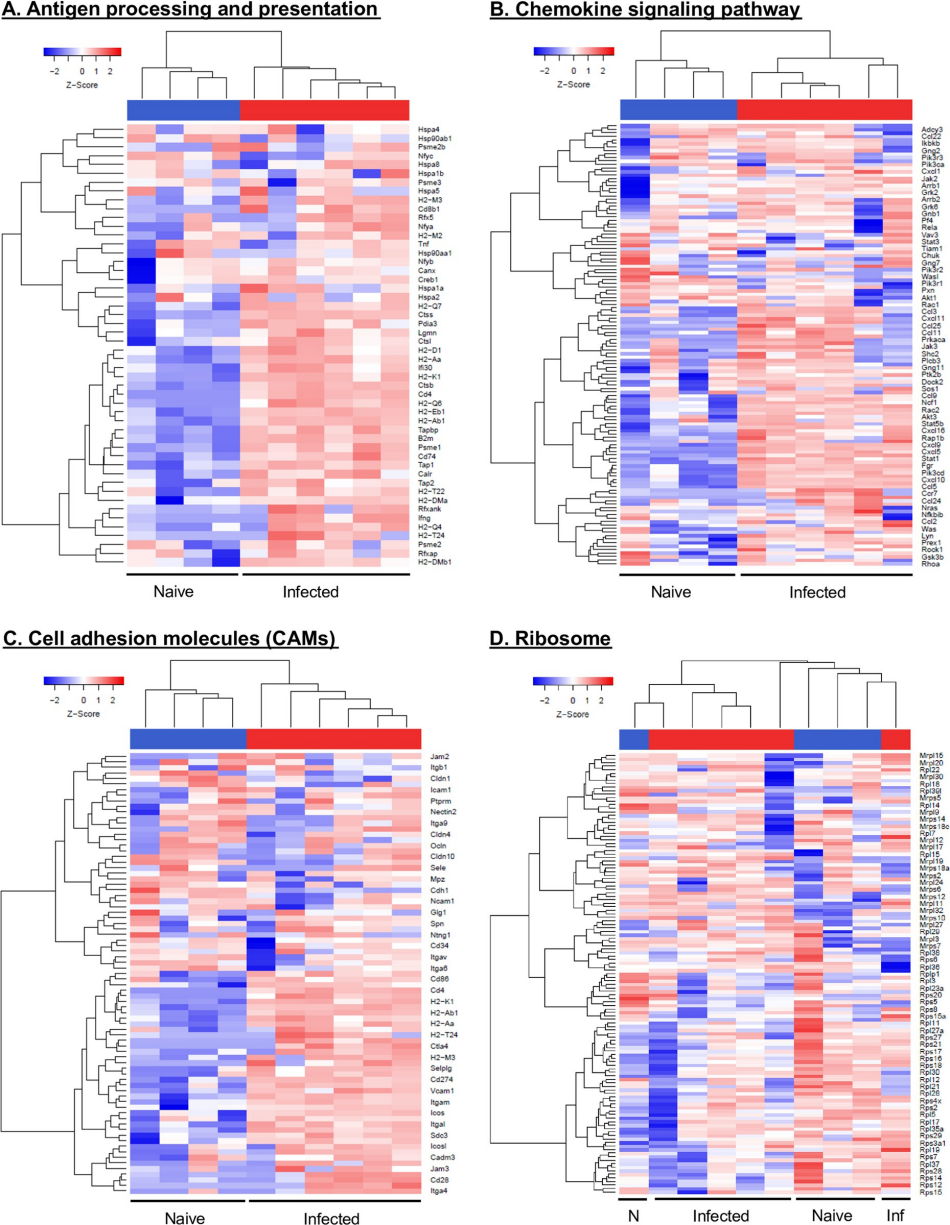
Heat map analysis of host transcriptional responses to *L*. *major* infection in vivo. Heat maps of DEGs in infected ears compared to naive controls. The DEGs involved in the host immune response pathways by KEGG enrichment analysis whether upregulated **(A, B, and C)** or downregulated **(D)** in the infected ears presented as heat maps. Hierarchical clustering of the expression profile was grouped according to functional categories. Heat maps indicate the FC of gene expression in *L*. *major*-infected ears >2-fold (red) or <2-fold (blue). Data are shown from 4 naïve control and 6 *L*. *major* infected mice.

Using hierarchical clustering analysis, we found that a large number of transcripts were increased in RNA abundance in the infected ears compared to naives (Fig 2A–2D). A heat map of the DEGs shows the expression profiles of infected and naive mice resulted in separate clusters (Fig 2). Hierarchical clustering reveals the host immune response to *L*. *major* infection is closely linked with DEGs encoding antigen processing and presentation pathway (*Cd4*, *H2-Q6*, *H2-M3*, *H2-Q4*, *Ifng*, *Cd8b1*, *H2-T22*, *Rfx5*, *Tap1*, *H2-Q7*), chemokine signaling (*Cxcl9*, *Ccl5*, *Ccr7*, *Cxcl10*, *Cxcl5*, *Cxcl16*, *Cxcl1*, *Fgr*, *Pik3cd*), and cell adhesion molecules (*Cd4*, *Itgam*, *Itgal*, *Ctla4*, *Icos Itga6*, *Cd274*, *Cd28*, *Cd86*, *Selplg*, *Vcam1*) (Fig 2A–2C). Additionally, we also noted the enrichment of DEGs for other immune network pathways which include cytokine-cytokine receptor interaction, phagosome, toll-like receptor signaling, and leukocyte trans-endothelial migration pathway (S2A–S2D Fig). In contrast, the biological processes downregulated with infection include ribosomal biogenesis (*Rpl3*, *Rpl37*, *Rps5*, *Rpl11*, *Rplp1*, *Rpl28*, *Rpl19*, *Rps28*, *Rps14*) (Fig 2D). Of note, the transcript results obtained from infected mice clustered together for antigen processing and presentation, chemokine signaling, and the cell adhesion molecules pathways (Fig 2A–2C), but transcripts from one mouse in each group clustered with the opposing experimental group for the ribosomal pathway (Fig 2D). Overall, these results demonstrate the host transcriptome undergoes reprogramming in the skin during *L*. *major* infection.

### scRNA-Seq reveals the cellular heterogeneity and altered transcriptomic profile of individual cell types during murine *L*. *major* infection in vivo

**Fig 3:**
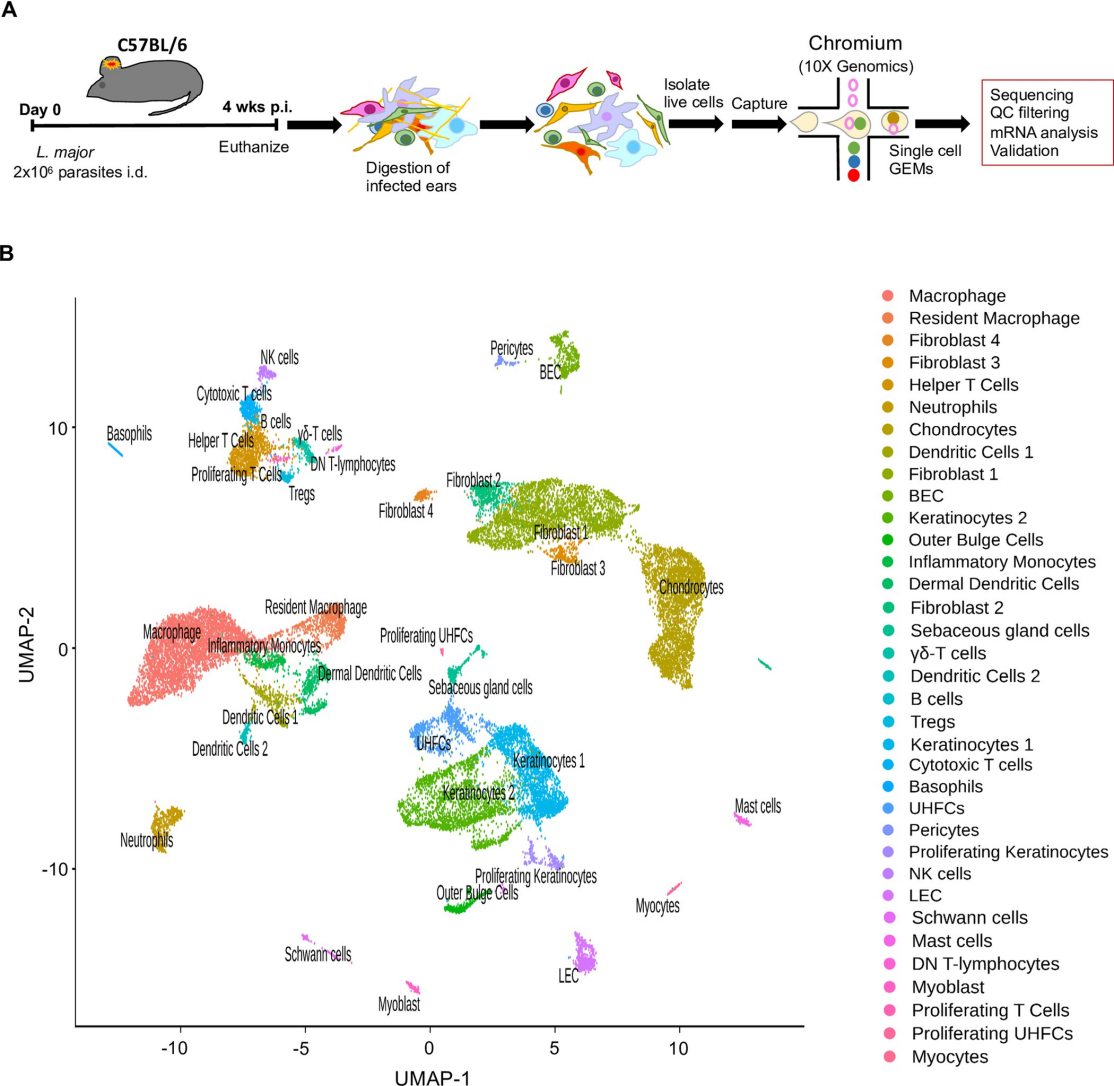
scRNA-Seq reveals cellular heterogeneity including distinct resident and recruited cell types in the skin following *L*. *major* infection. **(A)** C57BL/6 mice were infected or not with *L*. *major* parasites in the ear, and ears were digested to isolate RNA for scRNA-Seq analysis. Schematic of cell isolation, cell processing, capture by droplet-based device. **(B)** Uniform Manifold Approximation and Projection (UMAP) plot revealed cellular heterogeneity with 35 distinct clusters of cells identified and color-coded (both naive and infected groups combined). Seurat’s FindClusters function was used to identity each cell cluster and cell type designation is located to the right.

**Fig 4:**
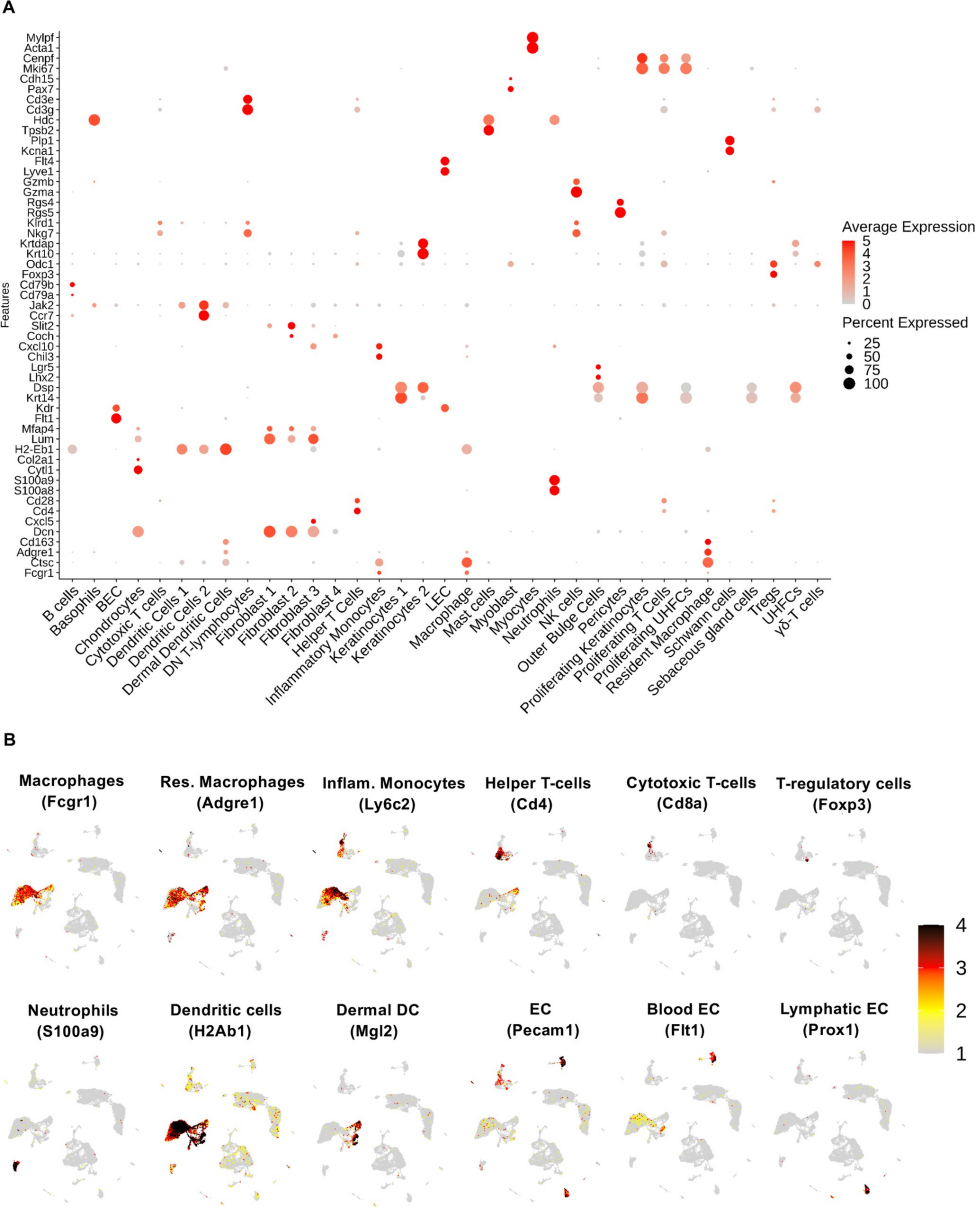
Cell type identification and cluster-specific gene expression. **(A)** Relative expression of 35 different cell type cluster-specific genes shown as dot plots with two genes per cluster. Dot size indicates the percentage of cells expressing each gene, the dot color indicates the expression level, and ordering is performed from low to high expressing cells. **(B)** Feature plots of expression distribution for selected cluster-specific genes used to define the cell types. Expression levels for each marker is color-coded and overlaid onto UMAP plot. Cells with the highest expression level are colored black.

The bulk RNA-Seq analysis revealed global changes in the transcriptional profile between infected mice and naive controls following *L*. *major* inoculation. To further investigate transcriptomic changes within individual cell types present in leishmanial lesions, scRNA-Seq was performed to provide a deeper understanding of how individual cells function in the tissue microenvironment. Single cells from the ears of infected and naive mice were bar-coded and sequenced using the droplet-based 10X Genomics Chromium platform (Fig 3A). After quality control assessment and filtering, the datasets were processed using Cell Ranger software. Unbiased hierarchical clustering using the Seurat R package provides single-cell transcriptional profiling with 26,558 cells and displayed the cellular heterogeneity which includes both resident and recruited cell types. Cell populations from 35 distinct cell types were defined using canonical markers from published literature and online databases (Fig 3B) [58]. The dot plot representing the cell type-specific canonical markers for each cell lineage used to distinguish the 35 distinct clusters is provided (Fig 4A). Here, it is to be noted that we couldn’t separate out the free parasites that are possibly being attached to the various cell types. Amongst the 35 cell types, we identified 16 cell types containing immune cells. Feature plots show the expression of cell type-specific canonical markers (in addition to the cell type-specific canonical markers in Fig 4A) for 12 clusters with corresponding cell types (Fig 4B). Additionally, a heatmap shows the canonical cell type markers from all the immune cell types along with blood endothelial cells (BECs) and lymphatic endothelial cells (LECs) (S3 Fig).

### Detection of *Leishmania major* transcripts in multiple cell types other than macrophages

**Fig 5:**
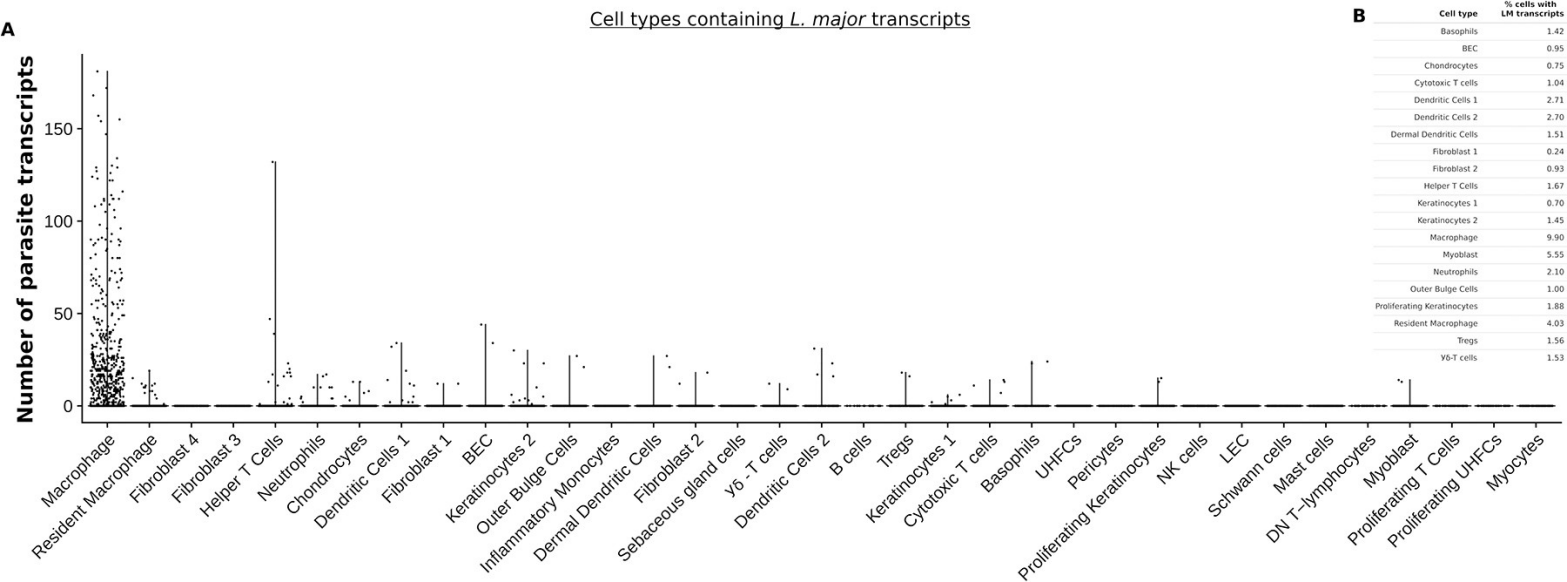
Presence of *L*. *major* transcripts in multiple cell types. **(A)** Differential expression of *L*. *major* parasite transcripts in 35 different cell types. Number of cells associated with parasite transcripts is shown in violin plots. **(B)** Table summarizes the percentages of individual cells associated with *L*. *major* transcripts.

We aligned the reads to *Leishmania major* (LM) reference genome to detect the presence of *Leishmania* transcripts in 35 different cell type clusters. Interestingly, the differential expression of LM transcripts from scRNA-Seq revealed 20 of 35 cell types have at least one LM transcript associated with that cell. As predicted, we found macrophages are the top immune cell type with about 10% of cells associated with LM transcripts (Fig 5A and 5B). We found at least 2% of other immune cell types are also associated with LM transcripts including resident macrophages, DCs, and neutrophils. At least 1% of the cells in CD4^+^ Th cells, CD8^+^ cytotoxic T cells, T regulatory cells, and basophils were also associated with detectable LM transcript (Fig 5A and 5B). Consistent with previous findings, we found fibroblasts and keratinocytes were also associated with LM transcripts [59]. Surprisingly, we also detected LM transcripts associated with >5% myoblasts, but not myocytes and almost 1% of the BECs. Overall, our data shows that multiple other cell types at the infection site are associated with LM transcripts suggesting other cell types maybe infected with parasites alongside monocytes and macrophages, which are the well-established primary host cells for *Leishmania* parasites.

### scRNA-Seq confirms the immune cell recruitment at the site of *L*. *major* infection in vivo

**Fig 6:**
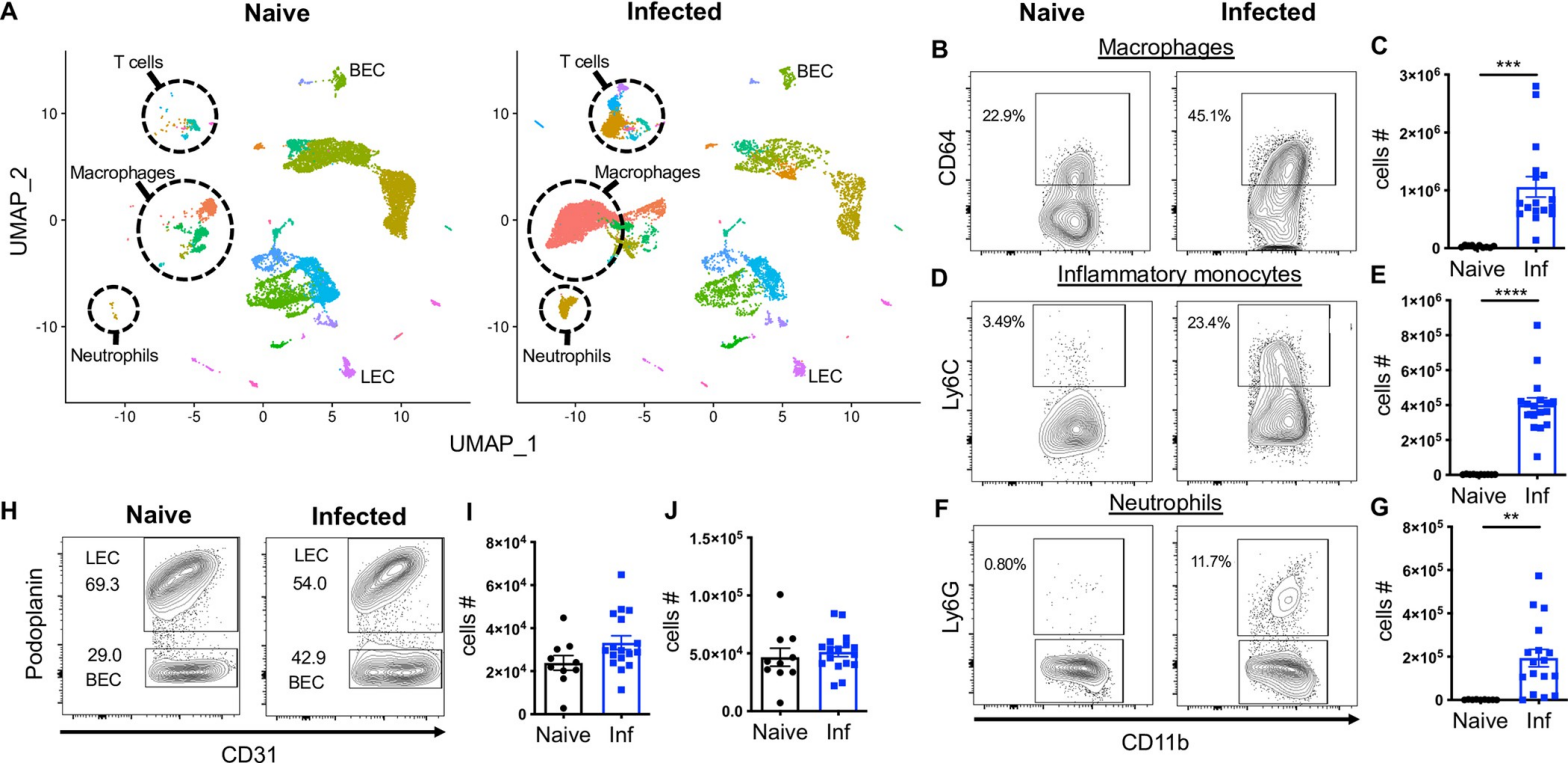
scRNA-Seq analysis reveals an enhanced immune cell recruitment to the inflamed tissue during *L*. *major* infection. **(A)** scRNA-Seq UMAP plots of naive and infected ears showing resident and recruited cell populations/clusters. **(B, D, F)** Representative flow cytometry plots showing the percentage of CD64^+^ macrophages (B), Ly6C^+^ inflammatory monocytes (D), and Ly6G^+^ neutrophils (F) of naive and infected ears at 4 week p.i. Cells were gated on total, live, singlets, CD45^+^ CD11b^+^ cells. **(C, E, G)** Cell numbers of CD64^+^ macrophages (C), inflammatory monocytes (E), neutrophils (G) from naive and infected ears. **(H-J)** ECs were gated on total, live, singlets, CD31^+^ cells. Dermal BECs and LECs were separated by podoplanin expression during FACS analysis. (H) Representative flow cytometry dot plots showing the percentages of BECs and LECs from naive and infected ears 4 week p.i. Corresponding cell numbers of BECs (I) and LECs (J) from naive and infected ears. Data are representative of at least two independent experiments involving 10–17 mice. Data are presented as mean ±SEM. **p < 0.*005*, ***p < 0.*0005*, ****p < 0.*0001*, unpaired *t*-test.

Murine *L*. *major* infection leads to the recruitment of immune cells such as neutrophils, inflammatory monocytes, and monocyte-derived macrophages to the site of infection. Particularly, inflammatory monocytes and both tissue-resident macrophages and monocyte-derived macrophages can serve as a replicative niche for the pathogen, as well as play a role in parasite control by killing the pathogen. BECs mediate immune cell recruitment to the infected and inflamed tissue and LECs promote immune cell migration away from the infected skin. Therefore, we speculate that immune cells and ECs participate in parasite control and/or immunopathology during CL. As a result, the remainder of the study focuses on 7 immune cell types along with the BEC and LEC clusters. Our UMAP projection displays 13,034 cells in naive ears and 13,524 cells in the infected ears (Fig 6A). Consistent with previous findings in CL, the UMAP plot confirms a significant recruitment of various immune cell types such as inflammatory monocytes, neutrophils, macrophages, dendritic cells, NK cells, and CD4^+^ and CD8^+^ T cells in the infected ears that are seen at higher frequencies compared to naive controls (Fig 6A). Concordant with our scRNA-Seq results, flow cytometric analysis detected a significant increase in the frequency and cell number of macrophages (Fig 6B and 6C), Ly6C^+^ inflammatory monocytes (Fig 6D and 6E) and neutrophils (Fig 6F and 6G) in infected ears compared to naive controls, while no significant alterations in the BEC or LEC populations were observed (Fig 6H–6J). Altogether, these data confirm the enhanced immune cell migration during *L*. *major* infection and transcriptional changes within these individual cell types were investigated.

### Differential gene expression of immune cell types during *L*. *major* infection

**Fig 7:**
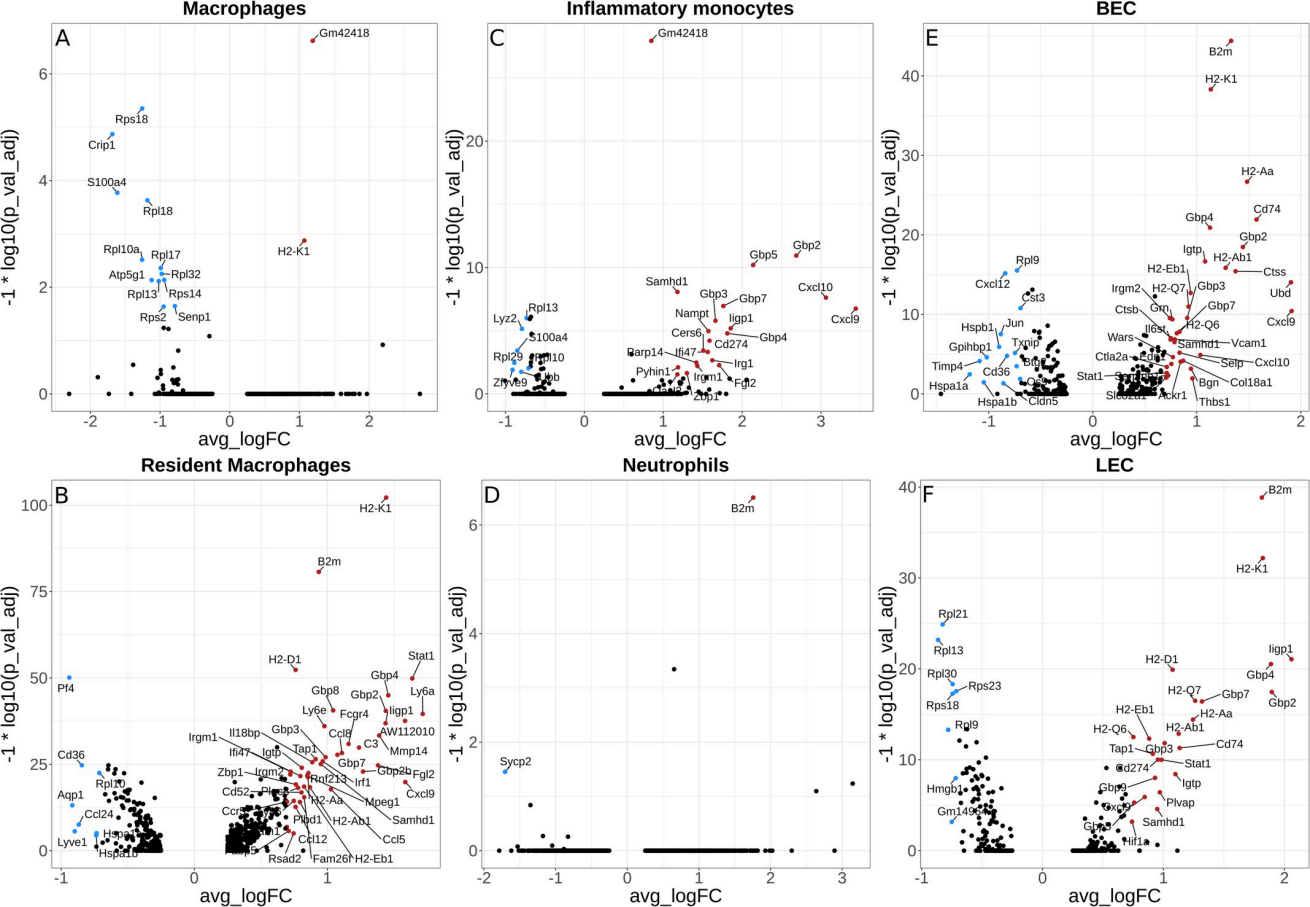
Differentially expressed genes in selected immune cell types during *L*. *major* infection. Volcano plot showing the DEGs in macrophages (A), resident macrophages (B), inflammatory monocyte (C), neutrophils (D), BECs (E), and LECs (F). Colored dots indicate genes at least 2 (natural log ~0.693) fold increased (red) or decreased (blue) in infected cells relative to naive cells with an adjusted p-value < 0.05.

To explore the transcriptional changes in a cell type-specific manner, the DEGs were compared between infected and naive mice within an individual cell type following *L*. *major* infection. A volcano plot showing DEGs for macrophages, resident macrophages, inflammatory monocytes, and neutrophils reveals several markers indicative of an increase in myeloid cells in leishmanial lesions (Fig 7A–7D). For instance, transcripts commonly elevated within the top 10 DEGs in myeloid cells and dendritic cells (DCs) include *B2m*, *H2-K1*, *Gbp2*, *ligp1*, whereas multiple ribosomal proteins showed reduced transcript abundance within the top 10 DEGs among myeloid cells and DCs (Tables 2 and 3 and S4 Fig). We found consistent elevation of various interferon-induced GTPases like guanylate binding protein (GBP) transcripts with *L*. *major* infection in macrophages (*Gbp2*) (Table 2A), resident macrophages (*Gbp4*, *Gbp8*, *Gbp2*) (Table 2B), inflammatory monocytes (*Gbp2*, *Gbp5*, *Gbp7*, *Gbp3*) (Table 3A), and DCs (*Gbp2*) (S4 Fig). Many of the transcriptomic differences detected in myeloid cells, such as elevated GBPs, were also found in EC populations. For instance, BECs expressed increased *Gbp4* and *Gbp2*, and LECs expressed increased *Gbp4*, *Gbp2*, and *Gbp7* upon infection (Table 4A–4B). Furthermore, we detected a significant abundance of both MHCI and MHCII transcripts in the infected ears in myeloid cells and ECs, which include *H2-K1*, *H2-Aa*, *H2-Ab1* in macrophages; *H2-K1*, *H2-D1* in resident macrophages; *H2-K1* in DCs, *H2-K1*, *H2-Aa*, *H2-Ab1* in BECs; and *H2-K1*, *H2-D1*, *H2-Q7*, *H2-Aa*, *H2-Ab1* in LECs (Tables 2 and 4 and S4 Fig). In contrast to myeloid cells, DCs, and ECs, we only detected few transcripts that are significantly elevated with infection in T cells including *B2m*, *Satb1*, *Gm42418*, *Gimap6* in CD4^+^ T cells and *Gm42418* in CD8^+^ T cells (S5 Fig).

**Table 2:** List of top 10 DEGs enriched in macrophages (A) and resident macrophages (B) between infected vs naïve controls.

(A) Macrophages			(B) Resident macrophages		
Gene name	Avg. log-fold change	Adj. p value	Gene name	Avg. log-fold change	Adj. p value
**Enriched**			**Enriched**		
*H2-K1*	1.203478	2.59E-30	*H2-K1*	1.441768	6.11E-103
*Gm42418*	1.013645	3.48E-30	*B2m*	0.935358	1.97E-81
*B2m*	0.798061	3.33E-28	*H2-D1*	0.761557	4.47E-53
*AW112010*	2.205139	1.15E-26	*Stat1*	1.637833	1.41E-50
*Fth1*	1.136905	2.54E-26	*Gbp4*	1.457725	1.05E-45
*Cxcl16*	1.778107	5.99E-22	*Gbp8*	1.043486	2.40E-41
*Gbp2*	1.141379	1.24E-21	*Gbp2*	1.43904	3.27E-41
*H2-Aa*	1.205499	1.62E-20	*Ly6a*	1.716371	2.56E-40
*H2-Ab1*	1.264542	9.46E-20	*AW112010*	1.583531	2.66E-38
*Mmp14*	1.784564	6.05E-19	*Iigp1*	1.437197	1.31E-37
**Depleted**			**Depleted**		
*Rpl32*	-1.2065	3.25E-34	*Pf4*	-0.93903	7.66E-51
*Rpl13*	-1.23205	2.53E-33	*Cd36*	-0.8449	1.88E-25
*Lyz2*	-1.48379	5.03E-32	*Rpl12*	-0.64508	1.92E-25
*Rpl18*	-1.22252	4.01E-30	*S100a6*	-0.59164	2.65E-24
*S100a4*	-1.6294	6.25E-30	*Rps25*	-0.64782	2.88E-24
*Rps18*	-1.21541	1.51E-28	*Rpl10*	-0.71332	3.09E-23
*Rps14*	-1.0183	1.73E-24	*Rnf213*	0.85734	3.50E-23
*Fau*	-1.03269	6.54E-23	*Rps12*	-0.5271	3.76E-23
*Ifitm3*	-1.30983	8.25E-23	*Rpl30*	-0.60024	4.48E-23
*Tpt1*	-0.83138	1.52E-22	*Rps18*	-0.56378	9.59E-21

**Table 3:** List of top 10 DEGs enriched in inflammatory monocytes (A) and neutrophils (B) clusters between infected vs naïve controls.

(A) Inflammatory monocytes			(B) Neutrophils		
Gene name	Avg. log-fold change	Adj. p value	Gene name	Avg. log-fold change	Adj. p value
**Enriched**			**Enriched**		
*Gm42418*	0.849004	1.16E-28	*B2m*	1.75856	3.13E-07
*Gbp2*	2.686188	1.14E-11	*Gm42418*	0.654377	0.000454
*Gbp5*	2.139579	6.38E-11	**Depleted**		
*Samhd1*	1.180168	8.55E-09			
*Cxcl10*	3.062718	2.43E-08	*Sycp2*	-1.70554	0.035225
*Gbp7*	1.761721	1.11E-07			
*Cxcl9*	3.440467	1.79E-07			
*Gbp3*	1.661975	1.62E-06			
*Iigp1*	1.855148	6.54E-06			
*Nampt*	1.57251	1.08E-05			
**Depleted**					
*Fau*	-0.67651	8.03E-07			
*Rpl13*	-0.73173	1.00E-06			
*Rps25*	-0.69169	1.18E-06			
*Lyz2*	-0.79057	7.16E-06			
*Rpl32*	-0.66904	1.75E-05			
*S100a4*	-0.85018	0.000375			
*Tpt1*	-0.47099	0.0008			
*Tmsb4x*	-0.51238	0.00088			
*Rpl9*	-0.66084	0.000907			
*Rpl39*	-0.55282	0.001096			

**Table 4:** List of top 10 DEGs enriched in BEC (A) and LEC (B) clusters following *L*. *major* infection.

(A) BEC			(B) LEC		
Gene name	Avg. log-fold change	Adj. p value	Gene name	Avg. log-fold change	Adj. p value
**Enriched**			**Enriched**		
*B2m*	1.330157	3.83E-45	*B2m*	1.81363722	1.46E-39
*H2-K1*	1.134934	4.90E-39	*H2-K1*	1.82143009	6.52E-33
*H2-Aa*	1.483775	2.02E-27	*Iigp1*	2.05863878	8.72E-22
*Cd74*	1.574517	1.16E-22	*Gbp4*	1.88908626	2.96E-21
*Gbp4*	1.127162	1.23E-21	*H2-D1*	1.07551012	1.24E-20
*Gbp2*	1.442935	3.36E-19	*Gbp2*	1.89596591	3.54E-18
*Igtp*	1.08015	2.13E-17	*H2-Q7*	1.26192971	3.25E-17
*H2-Ab1*	1.277568	1.37E-16	*Gbp7*	1.31819045	3.86E-17
*Ctss*	1.372639	3.69E-16	*H2-Aa*	1.24429821	3.81E-15
*Ubd*	1.905291	9.22E-15	*H2-Ab1*	1.1254732	1.38E-13
**Depleted**			**Depleted**		
*Rpl9*	-0.7283	2.97E-16	*Rpl21*	-0.82606	1.30E-25
*Cxcl12*	-0.84167	6.72E-16	*Rpl13*	-0.86409	6.46E-24
*Rpl21*	-0.58083	7.74E-14	*Rpl30*	-0.74324	4.82E-19
*Rpl13*	-0.62302	2.34E-13	*Rps23*	-0.71284	2.88E-18
*Cst3*	-0.69511	1.61E-11	*Rps18*	-0.74298	5.32E-18
*Rps14*	-0.43341	2.62E-09	*Rps11*	-0.62415	4.34E-14
*Rpl18*	-0.563	1.25E-08	*Rpl9*	-0.78012	5.04E-14
*Rps15a*	-0.51256	1.51E-08	*Rps25*	-0.68713	7.72E-13
*Rpl30*	-0.51597	1.72E-08	*Rps4x*	-0.54557	1.19E-12
*Rps18*	-0.51715	2.05E-08	*Rpl11*	-0.63287	3.41E-12

In addition, many chemokines were differentially modulated in myeloid cells and ECs with *L*. *major* infection (Fig 7). Specifically, *Cxcl9* was significantly elevated in macrophages, resident macrophages, inflammatory monocytes, DCs, BECs, and LECs following *L*. *major* infection (Figs 7 and S4). In contrast, we found significant downregulation of *Ccl24* in resident macrophages, *Cxcl12* in BECs, and *Ccl17* in DCs following *L*. *major* infection (Figs 7B and 7E and S4). Also important in immune cell recruitment, selectins (*Selp*, *Sele*) and adhesion molecules (*Vcam1)* were significantly upregulated in BECs with infection, while tight junction molecules like *Cldn5* were downregulated (Fig 7E). Of note, known canonical markers were significantly elevated with *L*. *major* infection including *Arg1*, *Nos2*, and *Pla2g7* in macrophages, *Fcgr4*, *C3*, and *Ccl8* in resident macrophages, and *Ifitm1*, *Syngr2*, *Cd200*, *Ccr7* in DCs; the complete list of DEGs that are enriched during *L*. *major* infection from these and other cell type clusters such as fibroblasts, keratinocytes, chondrocytes, sebaceous glands, basophils, upper hair follicle cells, pericytes, schwann cells, mast cells, myocytes and myoblasts can be found in Gene Expression Omnibus database with the GEO accession number—GSE181720.

### Characterization of upstream gene regulators and canonical pathways during *L*. *major* infection in vivo

Next, IPA analysis was performed to define the transcriptomic signature for each individual cell type at the site of *L*. *major* infection. IPA analysis of our scRNA-Seq data revealed several known and unknown canonical pathways, upstream regulators, and disease-based functional networks. Here, we present the transcripts that are significantly altered (adj. p value < 0.05) in macrophages, BECs, and LECs from infected ears compared to naive controls.

#### Upstream gene regulators

Our IPA analysis on macrophages, BECs, and LECs revealed potential transcription factors as well as transcriptional targets like anti- and pro-inflammatory genes. In macrophages, we observed 651 upstream regulators in total which include 38 upregulated and 17 downregulated gene regulators. We found cytokines like *IFNγ*, *IL-4*, *IL-13*, *IFNβ1*, *TNFα*, and transcriptional regulators such as *HIF1α*, *STAT1*, *CTCF*, *TP73*, *IRF1*, *MXD1*, *ATF4*, *SPI1* mediate macrophage activation upon infection (Table 5). In contrast, transcriptional regulators like *MLXIPL*, *MYC*, *TP53*, *MYCL*, *CEBPB*, *GATA1* were inhibited, and no cytokines were identified to downregulate macrophages activation following infection (Table 5). We identified 32 regulators were activated and 64 regulators were inhibited with infection in BECs. The upregulated cytokines activating BECs included *IFNγ*, *TNF*, *IL-2*, *IL-4*, while *IL-10* downregulates BEC activation (Table 6). Additionally, we found transcriptional regulators that are either activated (*IRF3*, *STAT1*, *IRF7*, *MXD1*) or inhibited (*MLXIPL*, *MYC*, *TRIM24*, *TP53*, *SIRT1*, *HSF1*, *MYCL*, *GLIS2*, *CEBPB*, *NFE2L2*) in BECs following infection (Table 6). Likewise, 212 upstream regulators were detected of LECs including 17 activated and 23 inhibited regulators. Corresponding to BECs, *IFNγ* increases LEC activation, while *IL-10* downregulates LEC activation upon infection (Table 7). In the LECs, we detected activated transcriptional regulators such as *IRF3*, *STAT1*, and *IRF7*; however, *MLXIPL*, *MYC*, *TRIM24*, *TP53*, *SIRT1*, *MYCL*, *TARDBP*, *STAT6*, *AIRE*, *LDB1*, *HNF4A*, and *NFE2L2* were inhibited in LECs comparing infected mice to naive controls (Table 7).

**Table 5:** List of top 5 upstream regulators identified in macrophages by IPA analysis for *L*. *major* infected ears.

Predicted activation state	Upstream regulator	Activation z-score	p-value of overlap	No of target molecules in dataset
**Cytokines**				
Activated	*IFNG*	4.44	9.87E-19	36
	*IL4*	2.433	1.31E-11	28
	*IL13*	2.713	8.71E-09	11
	*IFNβ1*	2.383	0.0000127	10
	*TNF*	3.364	0.0000372	12
Inhibited	Not detected	-	-	-
**Transcription regulators**				
Activated	*HIF1A*	2.075	1.62E-08	13
	*STAT1*	2.396	6.09E-08	12
	*CTCF*	2.236	5.05E-06	5
	*TP73*	2.333	5.53E-06	10
	*IRF1*	2.383	0.0000841	6
Inhibited	*MLXIPL*	-8.062	1.2E-88	65
	*MYC*	-7.457	3.77E-82	71
	*TP53*	-2.227	1.77E-19	51
	*MYCL*	-2.97	7.63E-07	9
	*CEBPB*	-2.54	0.0000174	16

**Note:** The activation Z-Scores (-8.062 to 4.44) and *p*-values were < 0.05.

**Table 6:** List of top 5 upstream regulators identified in BECs by IPA analysis for *L*. *major* infected ears.

Predicted activation state	Upstream regulator	Activation z-score	p-value of overlap	No of target molecules in dataset
***Cytokines***				
Activated	*IFNG*	3.684	5.19E-14	23
	*TNF*	2.99	5.79E-10	14
	*IL-2*	2.449	0.000141	7
	*IL-4*	2.219	0.00156	10
Inhibited	*IL-10*	-2.01	1.35E-11	14
**Transcription regulators**				
Activated	*IRF3*	3.065	2.11E-09	10
	*STAT-1*	2.377	1.37E-08	10
	*IRF7*	2.573	3.74E-07	7
	*MXD1*	2.0	0.00129	4
Inhibited	*MLXIPL*	-5.881	3.7E-48	36
	*MYC*	-5.207	3.54E-43	38
	*TRIM24*	-2.879	9.58E-12	12
	*TP53*	-3.877	1.8E-11	28
	*SIRT1*	-3.357	1.73E-10	15

**Note:** The activation Z-Scores (-5.881 to 3.684) and *p*-values were < 0.05.

**Table 7:** List of top 5 upstream regulators identified in LECs by IPA analysis for *L*. *major* infected ears.

Predicted activation state	Upstream regulator	Activation z-score	p-value of overlap	No of target molecules in dataset
**Cytokines**				
Activated	*IFNG*	3.644	1.62E-07	14
Inhibited	*IL-10*	-2.219	4.28E-07	9
**Transcription regulators**				
Activated	*STAT-1*	2.766	3.47E-12	12
	*IRF3*	3.266	9.08E-12	11
	*IRF7*	2.795	2.97E-09	8
Inhibited	*MLXIPL*	-6.97	2.47E-80	49
	*MYC*	-6.714	1.82E-68	49
	*SIRT1*	-4.338	8.72E-17	19
	*TRIM24*	-3.564	2.14E-14	13
	*MYCL*	-2.97	4.91E-10	9

**Note:** The activation Z-Scores (-6.97 to 3.644) and *p*-values were < 0.05.

### Canonical pathways

**Fig 8:**
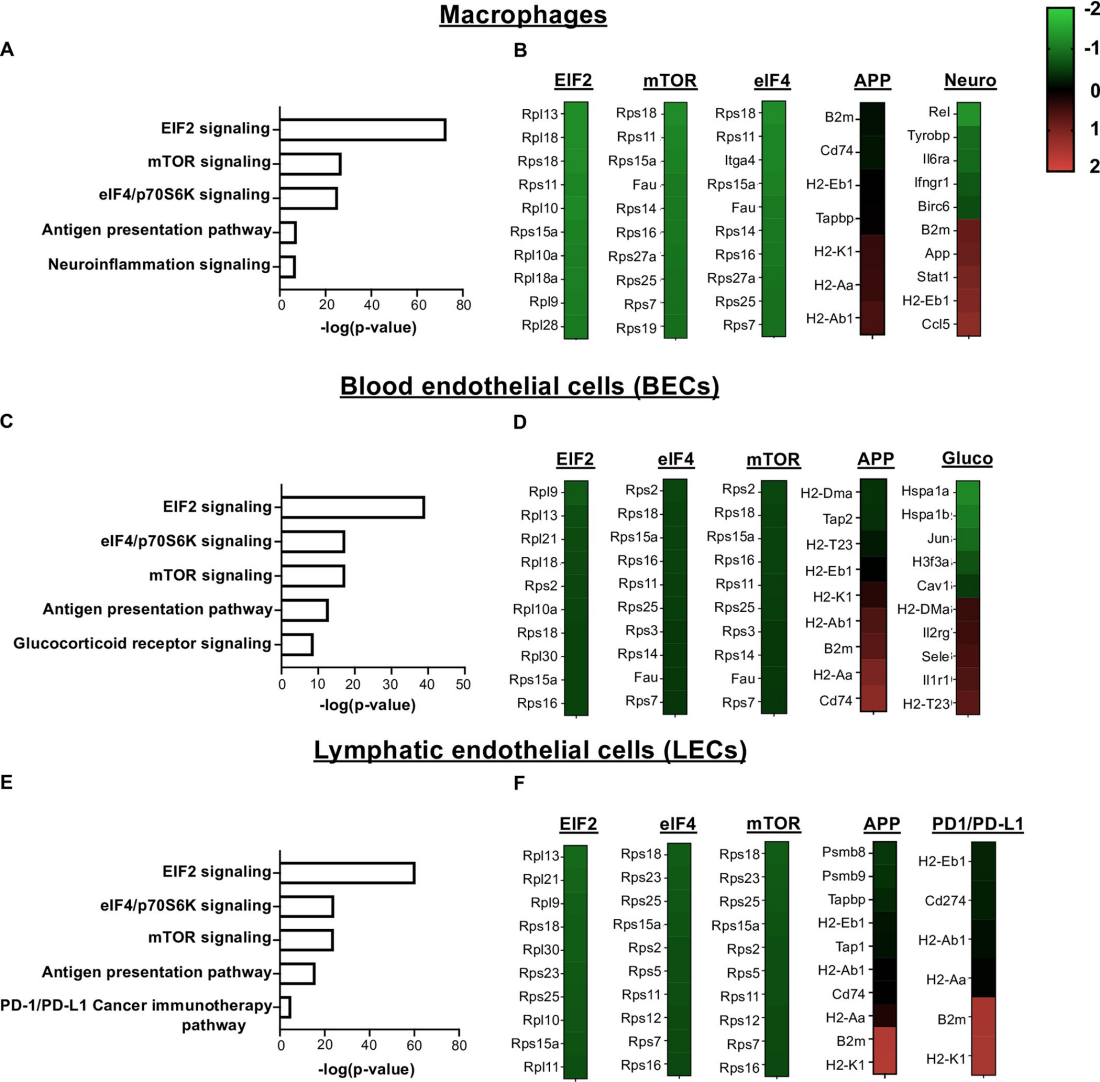
Signaling pathways and molecular networks within individual cell types predicted during *L*. *major* infection by Ingenuity Pathway Analysis. **(A-B)** Top 5 differentially regulated canonical pathways and their individual heat maps in macrophages following *L*. *major* infection. **(C-D)** Top 5 differentially regulated canonical pathways and their individual heat maps in BECs. **(E-F)** Top 5 differentially regulated canonical pathways and their individual heat maps in LECs. The color intensity represents the degree of expression. A red-green color scale was used to reflect the standardized gene expression with red representing high expression and green representing low expression. Cut-off values are adjusted p-value < 0.05.

**Fig 9:**
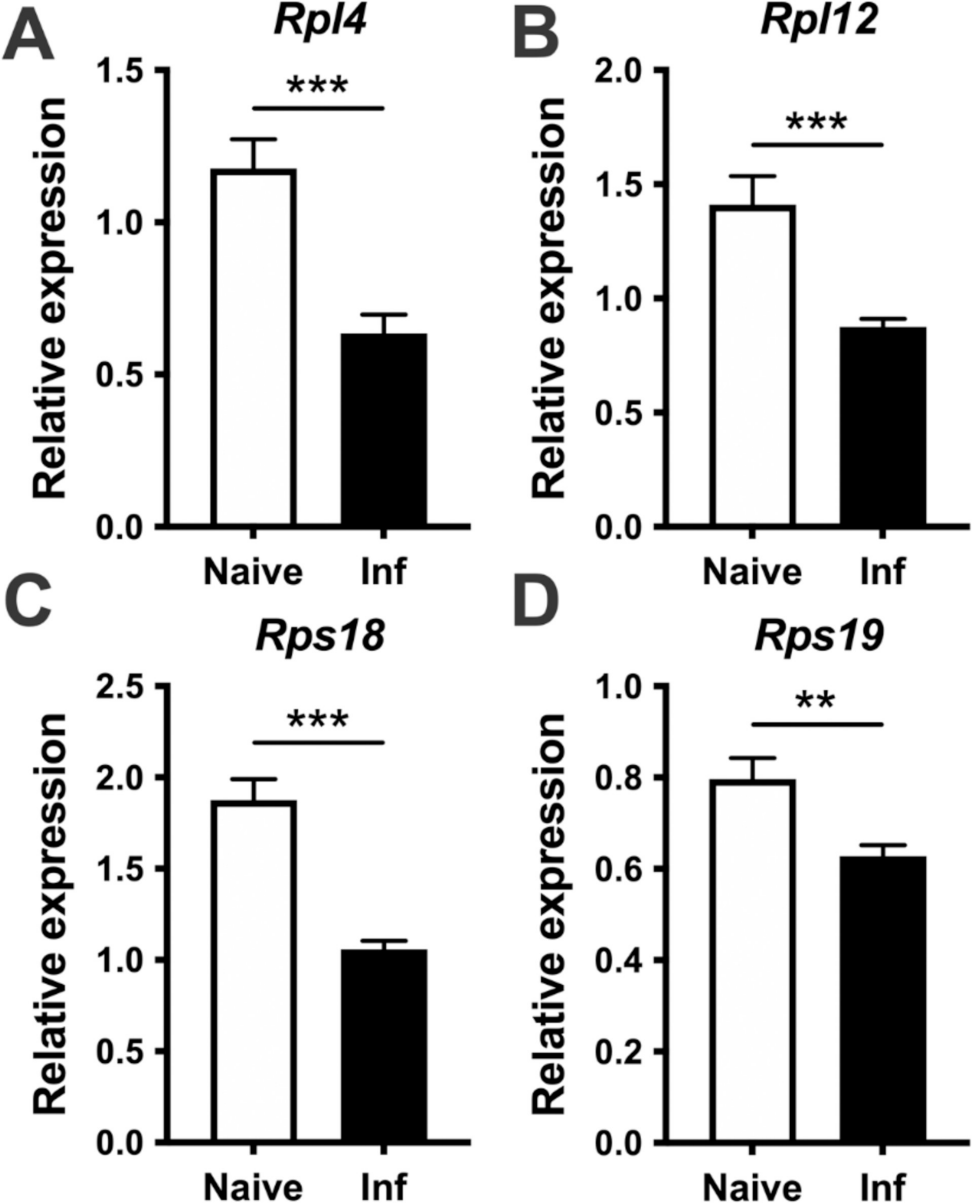
Ribosomal protein subunit large (Rpl) and small (Rps) transcripts are expressed at decreased levels at the site of *L*. *major* infection. C57BL/6 mice were infected with *L*. *major* parasites in the ear dermis, and ears were analyzed by quantitative real-time PCR at 4 wk p.i. for the expression of Rpl4 (**A**), Rpl12 (**B**), Rps18 (**C**), and Rps19 (**D**). Relative mRNA expression was normalized to the housekeeping gene RPS11 is presented as the mean ± SEM with 20–25 total mice. Data are representative of at least 4 independent experiments. * p<0.*05*, ** p<0.*005*, *** p<0.*0005 t*-test.

IPA analysis highlighted an unknown role for eukaryotic translation initiation factor 2 (EIF2) signaling which includes large ribosomal proteins (Rpl) and small ribosomal proteins (Rps) that had a significantly lower RNA abundance with infection compared to naive animals (Fig 8). Remarkably, the EIF2 pathway was the top downregulated pathway amongst multiple cell types including macrophages (Ranked as 1 amongst 377), BECs (Ranked as 1 amongst 257), and LECs (Ranked as 1 amongst 145) (Fig 8A and 8C and 8E). This was followed by an involvement of mTOR signalling and eIF4/p70S6K signalling in macrophages, BECs, and LECs from infected ears. Alongside the IPA pathway results, the expression of individual transcripts for each of the corresponding pathways is provided for macrophages (Fig 8B), BECs (Fig 8D), and LECs (Fig 8F). These data show the top 10 transcripts in the EIF2 signalling pathway in macrophages, BECs, and LECs, which includes many subunits of ribosomal proteins and the RNA abundance of these transcripts is mostly decreased in infected ears compared to naive controls (Fig 8B and 8D and 8F). In addition, we validated the relative expression of some of the Rpl and Rps transcripts by RT-PCR and found the significantly lowered expression of *Rpl4*, *Rpl12*, *Rps18*, *Rps19* during the infection compared to naïve animals (Fig 9A–9D). In contrast, the IPA analysis revealed the antigen presentation pathway was increased with infection, and this pathway was also a common feature of infection being the top elevated pathway for macrophages, BECs, and LECs (Fig 8B and 8D and 8F). The antigen presentation pathway was enriched in transcripts such as *B2m*, *Cd74*, *H2-K1*, *H2-Aa*, *H2-Ab1*, *H2-Eb1* that were elevated with *L*. *major* infection in macrophages, BECs, and LECs. In addition to macrophages, BECs, and LECs, we also noted that an involvement of mTOR signalling pathway is consistent among the other immune cell types apart of this study such as inflammatory monocytes, DCs, and CD4^+^ T cells, as mTOR signalling is top 5 in the list of pathways (S6 Fig). In summary, EIF2 signaling is the top downregulated pathway in BECs, LECs, macrophages, as well as other immune cell types from infected ears.

## Discussion

We conducted a comprehensive high-resolution transcriptomic analysis using both bulk and scRNA-Seq approaches to discover the global changes in the transcript expression that occurs following *L*. *major* infection in vivo. Through our bulk RNA-Seq analyses, we identified many differentially regulated novel transcripts in immune compartments that are related to host immune response pathways. We found significant enrichment of DEGs in the antigen processing and presentation pathway following *L*. *major* infection. Specifically, our data indicate that *L*. *major* infection upregulates many MHC molecules belonging to the antigen processing and presentation pathway along with inflammatory cytokines such as *IFNγ* following *L*. *major* infection. These findings are consistent with the well-established role of the Th1 immune response in parasite control; we confirm antigen presentation is a process associated with the host response to *Leishmania* infection [60–65]. Additionally, we noted the enrichment of DEGs specific for other host immune response pathways such as chemokine signalling, cell adhesion molecules, and cytokine-cytokine receptor interactions in infected ears. In contrast, our bulk RNA-Seq results revealed DEGs associated with the ribosomal pathway were downregulated following *L*. *major* infection. While the importance of the decrease in RNA abundance of transcripts that encode 40S and 60S ribosomal subunits has not been studied in *Leishmania*, these findings suggest cells at the site of infection are actively controlling translation and/or ribogenesis or undergoing a stress response similar to bacterial, viral infection and cancer [66–68]. Altogether, our bulk RNA-Seq results confirm a known role for the importance of antigen presentation and highlight an unknown feature of downregulating ribosomal subunits in CL.

Our scRNA-Seq analysis revealed many novel transcripts from various cell types that remain largely unexplored at the site of *L*. *major* infection. Through our scRNA-Seq data, we defined 35 distinct cell type populations by using canonical markers specific for different cell types including both resident and recruited cells following *L*. *major* infection. In agreement with previously published results [11,69], our scRNA-Seq analysis confirmed a significant increase in various immune cell types at the site of infection with recruited myeloid cells such as neutrophils, inflammatory monocytes, and monocyte-derived macrophages in lesions (Fig 6). The transcriptional signature of interferon-induced GTPases like GBPs was significantly upregulated in macrophages, resident macrophages, inflammatory monocytes, DCs, BECs, and LECs following *L*. *major* infection. These data suggest GBPs may play a protective role in both immune and nonimmune cells during *L*. *major* infection. GBPs are involved in controlling intracellular pathogen replication and specifically mediating the protection against intracellular pathogens such as *Listeria monocytogenes* and *Mycobacterium bovis* [70] [71]. Importantly, mice deficient in GBP genes are more susceptible to *Toxoplasma gondii* [72]. Moreover, GBPs restrict *L*. *donovani* growth in nonphagocytic cells such as murine embryonic fibroblasts in an IFNγ-dependent manner [73].

We detected several transcripts for chemokines in myeloid cells that are elevated with infection that may mediate myeloid cell accumulation at the site of infection. Similar to the transcriptomic findings of Carneiro et al., we detected increased RNA abundance of *Ccl5*, *Cxcl9* and *Cxcl10* with infection, whereas *Ccl2* and *Ccl7* was not detected in inflammatory monocytes and macrophage population in our data set [16]. However, unlike Carneiro et al., we did not separate infected from bystander inflammatory monocytes in our studies. CXCL9 and CXCL10 are monocyte-recruiting chemokines produced in response to IFNγ further supporting a potential role for Th1 immunity in recruiting permissive Ly6C^+^ CCR2^+^ monocytes during *Leishmania* infection [16]. Our results revealed a significant downregulation of *Ccl24* transcript in resident macrophages from infected ears compared to naive controls. A recent study demonstrated dermal tissue-resident macrophages shift toward a pro-inflammatory state in *L*. *major*-infected mice lacking IL-4/IL-13 from eosinophils, which is mainly regulated by CCL24 from resident macrophages [74]. Also, potentially mediating myeloid cell migration, we found BECs in infected skin express elevated transcripts for selectins (*Sele*, *Selp*) and adhesion molecules (*Vcam1*), while concomitantly downregulating transcripts responsible for junctional stability. Mice doubly deficient in E- and P-selectin develop significantly less inflammation following *L*. *major* infection [75]. Taken with our findings, these data suggest BECs play an active role during CL to recruit immune cells into the site of infection. Indeed, these scRNA-Seq data also correlate with our bulk RNA-Seq results which indicate a role of leukocyte trans-endothelial migration pathway in infected ears when compared to naive controls. Overall, our scRNA-Seq data reveals cells in leishmanial lesions exist in pro-inflammatory environment, but the actual host protective role of individual DEGs within each cell type requires further investigation.

We detected the presence of LM transcripts associated with multiple cell types including myeloid cells like macrophages, inflammatory monocytes, neutrophils, and DCs which are all known to harbor parasites [59,76,77]. We also detected LM transcripts associated with stromal cells such as fibroblasts and keratinocytes, which can be infected by parasites [78,79]. Surprisingly, we also found evidence of parasite transcripts associated with myoblasts, chondrocytes, and BECs which has not previously been reported. Importantly, the molecular techniques used in these studies cannot differentiate between living and dead parasites and we can only report cells associated with parasite transcripts. Therefore, subsequent studies will need to determine if parasites in other non-myeloid cells like myoblasts and BECs are viable and capable of replication and infection. Others have hypothesized *Leishmania* parasites might evade the host immune responses by seeking shelter in different non-macrophage cell types including fibroblasts and keratinocytes in addition to infection in myeloid cells which exhibit a more robust pro-inflammatory response against *Leishmania* parasites [80]. However, the presence of *L*. *major* transcripts from non-myeloid stromal cell types like myoblasts and BECs needs to be further explored to determine whether these cells can serve as safe havens for *L*. *major* parasites during chronic infection or provide a conduit for metastasis. It should be noted there are limitations to these findings as the current analysis cannot distinguish between cells with internalized parasites compared to cells with parasites that are co-purified with cells given free parasites are present in tissues and parasites could be attached to the outside surface of cells. Regardless, these results shed light on cell types previously not thought to harbor *Leishmania* parasites in the skin and provide an opportunity for new investigation. Lastly, future studies will compare the transcriptomic profiles of cells that are associated with LM transcripts with cells that are not are associated with LM transcripts.

Inflammatory monocytes and macrophages serve as replicative niches for the parasite as well as the major cell types responsible for parasite control. BECs play a crucial role in regulating immune cell entry into inflamed tissues and LECs participate in immune cell migration out of the lesions. As a result of the critical roles of these cell types during *L*. *major* infection, we focused on identifying the upstream regulators and canonical pathways for macrophages, BECs, and LECs. Surprisingly, the antigen processing and presentation pathway and EIF2 signaling were the most significant pathways in all three cell types (macrophages, BECs, and LECs) within infected ears. The antigen processing and presentation showed a positive activation z score indicating overall upregulation of the pathway, which is consistent with findings in human CL lesions [81]. Immunoproteasomes play a critical role in the immune response by degrading intracellular proteins to generate MHCI epitopes for effective antigen presentation [82]. While the increased expression of immunoproteasome genes in human lesions caused by *L*. *braziliensis* infection has been reported [81], our results reveal for the first time that LECs express higher levels of transcripts for immunoproteasomes (*psmb8* and *psmb9*), as well as transcripts involved in the antigen presentation pathway (*Tap1* and *Tapbp*) following *L*. *major* infection. These data suggest that ECs, and specifically LECs, may play an unknown role in antigen presentation during *L*. *major* infection, similar to viral infection and vaccination [82,83].

The EIF2 signaling pathway had the highest negative activation z score of all the pathways indicating overall downregulation during CL. To our knowledge, this is the first study highlighting EIF2 signaling as a novel candidate pathway for leishmaniasis. Eukaryotic initiation factor-2 (EIF2) is a GTP-binding protein, which initiates protein translation by delivering charged initiator met-tRNA onto the ribosome [84]. Upon subject to infection-induced cellular stress, EIF2 plays a significant role in attenuating translation initiation by phosphorylation of the alpha subunit of eIF2 leading to immediate shut-off of translation and activation of stress response genes [84]. Phosphorylation of eIF2α plays as a rate limiting step as it reduces active eIF2-GTP levels at translation initiation which ultimately results in a global reduction of protein synthesis [84,85]. Our data indicate that impaired EIF2 signaling is linked to the downregulation of many ribosomal subunits in macrophages, BECs, and LEC in *L*. *major*-infected ears (Fig 8). The known phenomenon of “protein shut off” has been well described at the molecular level for some viruses [86], but there is little to no evidence documenting this phenomenon during *L*. *major* infection. Generally, the enhanced host response to viral and bacterial infections depends on the upregulation of EIF2-mediated translational control, thereby reducing general protein synthesis [67,68,86]. However, we found EIF2 signaling was downregulated with *L*. *major* infection. Previously, it was demonstrated that *L*. *major* promotes its survival by downregulating macrophage protein synthesis, which is mainly mediated by host translation repressor 4E-BP1 [87]. Given the robust production of cytokines and chemokines in leishmanial lesions, global protein synthesis does not seem impacted by *L*. *major* infection in vivo, but careful analysis of EIF2 signaling has not been performed to date.

Alongside EIF2 signalling, the involvement of mTOR and eIF4/p70S6K signalling in macrophages, BECs, LECs, DCs and inflammatory monocytes in infected mice hints at an important role for hypoxia-induced oxidative stress at the site of *L*. *major* infected ears. Our results indicate the activation of metabolic gene targets like hypoxia-inducible factor (HIF-1α) in macrophages, which is consistent with our previous results and work by the Jantsch lab showing leishmanial lesions are hypoxic [9,88–90]. However, the consequence of hypoxia-induced oxidative stress and/or HIF-1α signaling is complex, and appears to be context dependent based on the cell type or *Leishmania* species as HIF-1α hampers DC function during VL, but enhances macrophage effector responses in both parasite control and orchestrating vascular remodeling in CL [9,10,89–92]. We speculate EIF2 signaling is downregulated as part of the stress response, potentially from hypoxia, but mechanism by which EIF2 signaling is impaired in multiple cell types and how that contributes to pathogen control or the pathogenesis of disease in CL is unknown. A caveat to our studies is that we only examined a single time point at 4 weeks p.i. following *L*. *major* inoculation. Over the course of infection, parasite burdens and lesion severity changes with time. The lesion microenvironment is dynamic and composed of different cell types at different time points following infection, especially as the inflammatory reaction subsides and the wound healing process takes over. However, these findings will inform subsequent studies examining the transcriptomic profiles of individual cell types at other time points following inoculation such as 1–2 weeks p.i. when the Th1 response is active but lesions are not present, at 8–10 week p.i. when lesions are healing, and at 12 weeks p.i. when lesions have resolved. Immune cells are recruited into lesions but our analysis also does not take into consideration how long a cell has resided in the lesion, being exposed to soluble mediators and the hypoxic microenvironment which could dramatically reprogram the transcriptomic signature of that cell.

Collectively, our transcriptome analysis not only provides the first comprehensive list of gene expression at single-cell resolution, but also highlights a previously unknown role of the highly conserved EIF2 signaling pathway in leishmaniasis. At the same time, our study has some limitations. This work was only performed in a resistant C57BL/6 mouse model, and it is not clear if the transcriptomic findings such as the decrease in EIF2 signaling and ribosomal transcripts are a general feature of CL. Future studies will need to determine if our findings will be replicated in a susceptible BALB/c mouse infected with *L*. *major*. Similarly, this study was only performed with *L*. *major* and it remains unknown if other healing and nonhealing CL species like *L*. *mexicana* or *L*. *amazonensis* will yield similar results. Regardless, future analysis by us and others utilizing these datasets will expand our knowledge on the complex immune and stromal cell networks and pathways participating in the host response to *Leishmania* infection.

## Materials and methods

### Ethics statement

All animal procedures were performed in accordance with the guidelines of the UAMS Institutional Animal Care and Use Committee (IACUC) under the animal protocol 4013.

### Animals

Female C57BL/6NCr mice were purchased from the National Cancer Institute. Mice were housed in the Division of Laboratory Animal Medicine at University of Arkansas for Medical Sciences (UAMS) under pathogen-free conditions and used for experiments between 6 and 8 weeks of age.

### Parasite infection in vivo

*Leishmania major* (WHO/MHOM/IL/80/Friedlin) strain was used. Parasites were maintained in vitro in Schneider’s Drosophila medium (Gibco) supplemented with 20% heat-inactivated FBS (Invitrogen), 2 mM L-glutamine (Sigma), 100 U/mL penicillin, and 100 mg/mL streptomycin (Sigma). Metacyclic stationary phase promastigotes were isolated from 4–5 day cultures by Ficoll density gradient separation (Sigma), as previously described [93]. For ear dermal infections, 2×10^6^ *L*. *major* promastigote parasites in 10 μL PBS (Gibco) were injected intradermally into the ear. Lesion development was monitored weekly by measuring ear thickness and lesion area with a caliper. Lesion volume was calculated. At 4-week post infection, ears were excised and enzymatically digested using 0.25 mg/mL Liberase (Roche) and 10 mg/mL DNase I (Sigma) in incomplete RPMI 1640 (Gibco) for 90 min at 37°C. After digesting, ears were minced manually to obtain cellular content in single cell suspensions. Parasite burdens were determined by limiting dilution assays (LDAs), as previously described[94].

### Bulk RNA-Seq: Sample preparation

The total RNA was isolated from the cell lysate of naive and infected ears by using Qiagen’s RNeasy plus mini kit according to the manufacturer’s instructions. The CTPR Genomics and Bioinformatics Core at the Arkansas Children’s Research Institute (ACRI) prepared sequencing libraries from RNA samples by use of the Illumina TruSeq Stranded mRNA Sample Preparation Kit v2. for sequencing on the NextSeq 500 platform using Illumina reagents. The quality and quantity of input RNA was determined using the Advanced Analytical Fragment Analyzer (AATI) and Qubit (Life Technologies) instruments, respectively. All samples with RQN (RNA quality number) values of 8.0 or above were processed for sequencing. Sequencing libraries were prepared by use of the TruSeq Stranded mRNA Sample Prep Kit (Illumina). Briefly, total RNA (500 ng) was subjected to polyA selection, then chemically fragmented and converted to single-stranded cDNA using random hexamer primed reverse transcription. The second strand was generated to create double-stranded cDNA, followed by fragment end repair and addition of a single A base on each end of the cDNA. Adapters were ligated to each fragment end to enable attachment to the sequencing flow cell. The adapters also contain unique index sequences that allow the libraries from different samples to be pooled and individually identified during downstream analysis. Library DNA was PCR amplified and enriched for fragments containing adapters at each end to create the final cDNA sequencing library. Libraries were validated on the Fragment Analyzer for fragment size and quantified by use of a Qubit fluorometer. Equal amounts of each library was pooled for sequencing on the NextSeq 500 platform using a high output flow cell to generate approximately 25 million 75 base reads per sample.

### Bulk RNA-Seq: Data analysis

Following demultiplexing, RNA reads were checked for sequencing quality using FastQC (http://www.bioinformatics.babraham.ac.uk/projects/fastqc) and MultiQC [95] (version 1.6). The raw reads were then processed according to Lexogen’s QuantSeq data analysis pipeline with slight modification. Briefly, residual 3’ adapters, polyA read through sequences, and low quality (Q < 20) bases were trimmed using BBTools BBDuk (version 38.52) (https://sourceforge.net/projects/bbmap/). The first 12 bases were also removed per the manufacture’s recommendation. The cleaned reads (> 20bp) were then mapped to the mouse reference genome (GRCm38/mm10/ensemble release-84.38/ GCA_000001635.6) using STAR [96] (version 2.6.1a), allowing up to 2 mismatches depending on the alignment length (e.g. 20-29bp, 0 mismatches; 30-50bp, 1 mismatch; 50–60+bp, 2 mismatches). Reads mapping to > 20 locations were discarded. Gene level counts were quantified using HTSeq (htseq-counts) [97] (version 0.9.1) (mode: intersection-nonempty).

Genes with unique Entrez IDs and a minimum of ~2 counts-per-million (CPM) in 4 or more samples were selected for statistical testing. This was followed by scaling normalization using the trimmed mean of M-values (TMM) method [98] to correct for compositional differences between sample libraries. Differential expression between naive and infected ears was evaluated using limma voomWithQualityWeights [99] with empirical bayes smoothing. Genes with Benjamini & Hochberg [100] adjusted p-values ≤ 0.05 and absolute fold-changes ≥ 1.5 were considered significant.

Gene Set Enrichment Analysis (GSEA) was carried out using Kyoto Encyclopedia of Genes and Genomes (KEGG) pathway databases and for each KEGG pathway, a p-value was calculated using hypergeometric test. Cut-off of both *p* < 0.05 and adjusted p-value/FDR value < 0.05 was applied to identify enriched KEGG pathways. DEGs that are more than 1.5-fold in *L*. *major*-infected ears relative to uninfected controls were used as input, with upregulated and downregulated genes considered separately. Subsequently, the heat maps were generated using these genes with complex Heatmap. All analyses and visualizations were carried out using the statistical computing environment R version 3.6.3, RStudio version 1.2.5042, and Bioconductor version 3.11. The raw data from our bulk RNA-Seq analysis were deposited in Gene Expression Omnibus (GEO accession number—GSE185253).

### scRNA-Seq sample preparation

The Genomics and Bioinformatics Core at the Arkansas Children’s Research Institute (ACRI) prepared NGS libraries from fresh single-cell suspensions using the 10X Genomics NextGEM 3’ assay for sequencing on the NextSeq 500 platform using Illumina SBS reagents. The quantity and viability of cells input to the assay was determined using Trypan Blue exclusion under 10X magnification. Library quality was assessed with the Advanced Analytical Fragment Analyzer (Agilent) and Qubit (Life Technologies) instruments.

### scRNA-Seq data analysis

Demultiplexed fastq files generated by the UAMS Genomics Core were analyzed with the 10X Genomics Cell Ranger alignment and gene counting software, a self-contained scRNA-Seq pipeline developed by 10X Genomics. The reads are aligned to the mm10 and *Leishmania major* reference transcriptomes using STAR and transcript counts are generated [96,101].

The raw counts generated by *cellranger count* were further processed using the R package *Seurat* [102,103]. Low quality cells, potential cell doublets, and cells with high percentage of mitochondrial genes were filtered out of the data. We filtered cells that have unique feature counts over more the 75^th^ percentile plus 1.5 times the interquartile range (IQR) or less than the 25^th^ percentile minus 1.5 time the IQR. Additionally, we filtered cells with mitochondrial counts falling outside the same range with respect to mitochondrial gene percentage. Following filtering the counts all 8 sequencing runs were merged. The counts are then normalized using the LogNormalize method, which normalizes the feature expression measurements for each cell by the total expression, multiplies this by a scale factor (10,000 by default), and log-transforms the result. Next, the 2000 highest variable features are selected. The data is then scaled, and linear regression is included to remove variation associated with percent mitochondria and cell cycle status. Principle component analysis (PCA) is performed on the scaled data. A JackStraw procedure was implemented to determine the significant PCA components that have a strong enrichment of low p-value features.

A graph-based clustering approach is applied [104]. Briefly, these methods embed cells in a graph structure—for example a K-nearest neighbor (KNN) graph, with edges drawn between cells with similar feature expression patterns, and then attempt to partition this graph into highly interconnected ‘quasi-cliques’ or ‘communities’. t-distributed stochastic neighbor embedding (tSNE) and Uniform Manifold Approximation and Projection (UMAP) [105] are non-linear dimensional reduction techniques used to visualize and explore the results and are performed using Seurat. Seurat *FindNeighbors* and *FindClusters* functions are optimized to label clusters based on the visual clustering in the projections. Seurat *FindAllMarkers* function finds markers that define clusters by differential expression. It identifies positive markers of a single cluster compared to all other cells and outputs the differential expression results. These markers are compared to known markers of expected cell types and results from previous single-cell transcriptome studies in order to assign appropriate cell type labels. Cell type determinations were made by manually expecting these results and some clusters were combined if their expression was found to be similar. Differential expression analysis is performed using MAST, a GLM-framework that treats cellular detection rate as a covariate [106]. The raw data from our scRNA-Seq analysis were deposited in Gene Expression Omnibus (GEO accession number—GSE181720).

### Ingenuity Pathway Analysis (IPA)

QIAGEN’s Ingenuity Pathway Analysis (IPA, QIAGEN Redwood City, www.qiagen.com/ingenuity) was utilized to investigate the DEGs at the level of biochemical pathways and molecular functions. We submitted our DEGs to functional analysis with IPA, and IPA provided canonical pathways, diseases and function, and upstream regulators based on the experimentally observed cause-effect relationships related to transcription, expression, activation, molecular modification, etc. Z-score analyses are used to assess the match between observed and predicted up and down regulation patterns allowing for Bayesian scoring of the results.

### Flow cytometry analysis with statistics

The recruitment of immune cells during *L*. *major* infection was analyzed by flow cytometry. Cells from both naive and infected ears were incubated with fixable Aqua dye (Invitrogen) to assess cell viability, and cells were treated with FcγR blocking reagent (Bio X Cell) prior to staining for the following markers: anti-CD45-AF700 (clone 30-F11), anti-Ly6C-PerCP-Cy5.5 (clone HK1.4), and anti-Ly6G-eFlour 450 (clone 1A8) were purchased from eBiosciences; anti-CD64-BV711 (clone X54-5/7.1), anti-CD11b-BV605 (clone M1/70), anti-CD31-AF488 (390), and anti-podoplanin-PE/Dazzle 594 (clone 8.1.1) were purchased from BioLegend. Cells were acquired using an LSR II Fortessa flow cytometer (BD Biosciences) and analyzed using FlowJo software version 10.2 (Tree Star). All statistical analyses were performed using Prism version 8.0 (GraphPad Software, Inc.). Comparisons between groups were performed using the two-tailed Students unpaired *t*-test.

## Supporting information

S1 Fig Lesion progression over time. C57BL/6 mice were infected with 2x10^6^ *L*. *major* metacyclic promastigote parasites intradermally in the ear and lesion development was monitored over time. Data are pooled from 4 experiments (n = 30) and shown as +SEM.
(TIF)

S2 Fig Heat map analysis showing transcriptional responses from other immune-related pathways during *L*. *major* infection in vivo. The DEGs involved in the other host immune response pathways by KEGG enrichment analysis (A, B, C and D) in the infected ears compared to naïve mice presented as heat maps. Hierarchical clustering of the expression profile was grouped according to functional categories. Heat maps indicate the FC in *L*. *major* infected ear gene expression >2-fold (red) or <2-fold (blue).
(TIF)

S3 Fig Differentially expressed genes in selected immune cell types during *L*. *major* infection. Heat map showing the three highly expressed genes for at least 14 immune cell clusters that were selected along with BECs and LECs. Each column represents a single cell and each row represents an individual gene. Three marker genes per cluster was color-coded and shown on the left. Yellow indicates maximum gene expression and purple indicates no expression in scaled log-normalized unique molecular identifier counts.
(TIF)

S4 Fig Differentially expressed genes in DCs during *L*. *major* infection. Volcano plot showing the DEGs in dendritic cells (DC1 and DC2) and list includes the top DEGs enriched in DCs following *L*. *major* infection. Colored dots indicate genes at least 2 (natural log ~0.693) fold increased (red) or decreased (blue) in infected cells relative to naïve cells with an adjusted p-value < 0.05.
(TIF)

S5 Fig Differentially expressed genes in CD4^+^ and CD8^+^ T cells during *L*. *major* infection. Volcano plot showing the DEGs in CD4^+^ and CD8^+^ T cells and list includes the top DEGs enriched in CD4^+^ and CD8^+^ T cells following *L*. *major* infection. Colored dots indicate genes at least 2 (natural log ~0.693) fold increased (red) or decreased (blue) in infected cells relative to naïve cells with an adjusted p-value < 0.05.
(TIF)

S6 Fig IPA predicted the role of mTOR signaling in other immune cell types during *L*. *major* infection by. **(A-C)** Differentially regulated canonical pathways in DCs (A), inflammatory monocytes (B), CD4^+^ T cells (C) following *L*. *major* infection.
(TIF)

S1 Table Ranked order list of all transcripts revealed by bulk RNA-Seq analysis. (XLSX)

S2 Table DEGs enriched for top 20 KEGG disease pathways. (DOCX)

## References

[1] Burza S, Croft SL, Boelaert M. Leishmaniasis. Lancet. 2018;392: 951–970. doi: 10.1016/S0140-6736(18)31204-2 30126638

[2] Zhang WW, Karmakar S, Gannavaram S, Dey R, Lypaczewski P, Ismail N, et al. A second generation leishmanization vaccine with a markerless attenuated Leishmania major strain using CRISPR gene editing. Nat Commun. 2020;11: 1–14. doi: 10.1038/s41467-020-17154-z 32651371 PMC7351751

[3] Arenas R, Torres-Guerrero E, Quintanilla-Cedillo MR, Ruiz-Esmenjaud J. Leishmaniasis: A review. F1000Research. 2017;6: 1–15. doi: 10.12688/f1000research.11120.1 28649370 PMC5464238

[4] Reithinger R. Cutaneous leishmaniasis. Lancet Infect Dis. 2007;146: 581–96. doi: 10.1016/j.annder.2019.02.002 17714672

[5] Terabe M, Kuramochi T, Ito M, Hatabu T, Sanjoba C, Chang KP, et al. CD4+ cells are indispensable for ulcer development in murine cutaneous leishmaniasis. Infect Immun. 2000;68: 4574–4577. doi: 10.1128/IAI.68.8.4574-4577.2000 10899857 PMC98378

[6] Pereira L de OR, Moreira RB, de Oliveira MP, Reis S de O, de Oliveira Neto MP, Pirmez C. Is Leishmania (Viannia) braziliensis parasite load associated with disease pathogenesis? Int J Infect Dis. 2017. doi: 10.1016/j.ijid.2017.01.036 28167253

[7] Gonzalez-Lombana C, Gimblet C, Bacellar O, Oliveira WW, Passos S, Carvalho LP, et al. IL-17 Mediates Immunopathology in the Absence of IL-10 Following Leishmania major Infection. PLoS Pathog. 2013;9. doi: 10.1371/journal.ppat.1003243 23555256 PMC3605236

[8] Bittencourt AL, Barral A. Evaluation of the histopathological classifications of American cutaneous and mucocutaneous leishmaniasis. Memórias do Instituto Oswaldo Cruz. 1991. pp. 51–56. doi: 10.1590/s0074-02761991000100009 1842401

[9] Weinkopff T, Roys H, Bowlin A, Scott P. Leishmania infection induces macrophage vascular endothelial growth factor A production in an ARNT/HIF-dependent manner. Infect Immun. 2019. doi: 10.1128/IAI.00088-19 31451620 PMC6803331

[10] Bowlin A, Roys H, Wanjala H, Bettadapura M, Venugopal G, Surma J. Hypoxia-Inducible Factor Signaling in Macrophages Promotes.: 1–16. 10.1128/IAI.00124-21 PMC8281282 34031127

[11] Scott P, Novais FO. Cutaneous leishmaniasis: Immune responses in protection and pathogenesis. Nat Rev Immunol. 2016;16: 581–592. doi: 10.1038/nri.2016.72 27424773

[12] Soong L, Chang CH, Sun J, Longley BJ, Ruddle NH, Flavell RA, et al. Role of CD4+ T cells in pathogenesis associated with Leishmania amazonensis infection. J Immunol. 1997;158: 5374–83. Available: http://www.ncbi.nlm.nih.gov/pubmed/9164958 9164958

[13] Scorza BM, Carvalho EM, Wilson ME. Cutaneous manifestations of human and murine leishmaniasis. International Journal of Molecular Sciences. 2017. doi: 10.3390/ijms18061296 28629171 PMC5486117

[14] Romano A, Carneiro MBH, Doria NA, Roma EH, Ribeiro-Gomes FL, Inbar E, et al. Divergent roles for Ly6C+CCR2+CX3CR1+inflammatory monocytes during primary or secondary infection of the skin with the intra-phagosomal pathogen Leishmania major. PLoS Pathog. 2017;13. doi: 10.1371/journal.ppat.1006479 28666021 PMC5509374

[15] Glennie ND, Volk SW, Scott P. Skin-resident CD4+T cells protect against Leishmania major by recruiting and activating inflammatory monocytes. PLoS Pathog. 2017;13. doi: 10.1371/journal.ppat.1006349 28419151 PMC5409171

[16] Carneiro MB, Lopes ME, Hohman LS, Romano A, David BA, Kratofil R, et al. Th1-Th2 Cross-Regulation Controls Early Leishmania Infection in the Skin by Modulating the Size of the Permissive Monocytic Host Cell Reservoir. Cell Host Microbe. 2020;27: 752–768.e7. doi: 10.1016/j.chom.2020.03.011 32298657

[17] Liew FY, Millott S, Parkinson C, Palmer RMJ, Moncada S. Macrophage killing of Leishmania parasite in vivo is mediated by nitric oxide from F Y Liew, S Millott, C Parkinson, R M Palmer and S Why The JI? • Rapid Reviews! 30 days * from submission to initial decision • No Triage! Every submission reviewed b. J Immunol. 1990;144. 2351828

[18] Müller AJ, Aeschlimann S, Olekhnovitch R, Dacher M, Späth GF, Bousso P. Photoconvertible pathogen labeling reveals nitric oxide control of leishmania major infection in vivo via dampening of parasite metabolism. Cell Host Microbe. 2013;14: 460–467. doi: 10.1016/j.chom.2013.09.008 24139402

[19] Terrazas C, Varikuti S, Oghumu S, Steinkamp HM, Ardic N, Kimble J, et al. Ly6Chi inflammatory monocytes promote susceptibility to Leishmania donovani infection. Sci Rep. 2017;7: 1–10. doi: 10. doi:10.1038/s41598-017-14935-3 29089636 10.1038/s41598-017-14935-3 PMC5665970

[20] Laskay T, Van Zandbergen G, Solbach W. Neutrophil granulocytes—Trojan horses for Leishmania major and other intracellular microbes? Trends Microbiol. 2003;11: 210–214. doi: 10.1016/s0966-842x(03)00075-1 12781523

[21] Ribeiro-Gomes FL, Sacks D. The influence of early neutrophil-Leishmania interactions on the host immune response to infection. Front Cell Infect Microbiol. 2012;2: 59. doi: 10.3389/fcimb.2012.00059 22919650 PMC3417510

[22] Peters NC, Egen JG, Secundino N, Debrabant A, Kimblin N, Kamhawi S, et al. In vivo imaging reveals an essential role for neutrophils in leishmaniasis transmitted by sand flies. Science (80-). 2008. doi: 10.1126/science.1159194 18703742 PMC2606057

[23] León B, López-Bravo M, Ardavín C. Monocyte-Derived Dendritic Cells Formed at the Infection Site Control the Induction of Protective T Helper 1 Responses against Leishmania. Immunity. 2007;26: 519–531. doi: 10.1016/j.immuni.2007.01.017 17412618

[24] Charmoy M, Brunner-Agten S, Aebischer D, Auderset F, Launois P, Milon G, et al. Neutrophil-derived CCL3 is essential for the rapid recruitment of dendritic cells to the site of Leishmania major inoculation in resistant mice. PLoS Pathog. 2010;6. doi: 10.1371/journal.ppat.1000755 20140197 PMC2816696

[25] Lai GN, Hsu A, Mandell MA, Roediger B, Hoeller C, Mrass P, et al. Migratory dermal dendritic cells act as rapid sensors of protozoan parasites. PLoS Pathog. 2008;4. doi: 10.1371/journal.ppat.1000222 19043558 PMC2583051

[26] Formaglio P, Alabdullah M, Siokis A, Handschuh J, Sauerland I, Fu Y, et al. Nitric oxide controls proliferation of Leishmania major by inhibiting the recruitment of permissive host cells. Immunity. 2021;54: 2724–2739.e10. doi: 10.1016/j.immuni.2021.09.021 34687607 PMC8691385

[27] de Castro Neto AL, da Silveira JF, Mortara RA. Comparative Analysis of Virulence Mechanisms of Trypanosomatids Pathogenic to Humans. Front Cell Infect Microbiol. 2021;11: 1–10. doi: 10.3389/fcimb.2021.669079 33937106 PMC8085324

[28] Arango Duque G, Jardim A, Gagnon É, Fukuda M, Descoteaux A. The host cell secretory pathway mediates the export of Leishmania virulence factors out of the parasitophorous vacuole. PLoS Pathog. 2019;15: 1–25. doi: 10.1371/journal.ppat.1007982 31356625 PMC6687203

[29] Reiner SL, Zheng S, Wang Z-E, Stowring L, Locksley RM. Leishmania Promastigotes Evade Interleukin 12 (IL-12) Induction by Macrophages and Stimulate a Broad Range of Cytoklnes from CD4 + T Cells during Initiation of Infection. Available: http://rupress.org/jem/article-pdf/179/2/447/1395232/447.pdf doi: 10.1084/jem.179.2.447 7905017 PMC2191353

[30] Matheoud D, Moradin N, Bellemare-Pelletier A, Shio MT, Hong WJ, Olivier M, et al. Leishmania evades host immunity by inhibiting antigen cross-presentation through direct cleavage of the SNARE VAMP8. Cell Host Microbe. 2013. doi: 10.1016/j.chom.2013.06.003 23870310

[31] Postat J, Olekhnovitch R, Lemaître F, Bousso P. A Metabolism-Based Quorum Sensing Mechanism Contributes to Termination of Inflammatory Responses. Immunity. 2018;49: 654–665.e5. doi: 10.1016/j.immuni.2018.07.014 30266340

[32] Heyde S, Philipsen L, Formaglio P, Fu Y, Baars I, Höbbel G, et al. CD11c-expressing Ly6C+CCR2+ monocytes constitute a reservoir for efficient Leishmania proliferation and cell-to-cell transmission. PLoS Pathog. 2018;14: 1–30. doi: 10.1371/journal.ppat.1007374 30346994 PMC6211768

[33] Olivier M, Gregory DJ, Forget G. Subversion mechanisms by which Leishmania parasites can escape the host immune response: A signaling point of view. Clin Microbiol Rev. 2005;18: 293–305. doi: 10.1128/CMR.18.2.293-305.2005 15831826 PMC1082797

[34] Kaye P, Scott P. Leishmaniasis: Complexity at the host-pathogen interface. Nat Rev Microbiol. 2011;9: 604–615. doi: 10.1038/nrmicro2608 21747391

[35] Holzer TR, McMaster WR, Forney JD. Expression profiling by whole-genome interspecies microarray hybridization reveals differential gene expression in procyclic promastigotes, lesion-derived amastigotes, and axenic amastigotes in Leishmania mexicana. Mol Biochem Parasitol. 2006;146: 198–218. doi: 10.1016/j.molbiopara.2005.12.009 16430978

[36] Leifso K, Cohen-Freue G, Dogra N, Murray A, McMaster WR. Genomic and proteomic expression analysis of Leishmania promastigote and amastigote life stages: The Leishmania genome is constitutively expressed. Mol Biochem Parasitol. 2007;152: 35–46. doi: 10.1016/j.molbiopara.2006.11.009 17188763

[37] McNicoll F, Drummelsmith J, Müller M, Madore É, Boilard N, Ouellette M, et al. A combined proteomic and transcriptomic approach to the study of stage differentiation in Leishmania infantum. Proteomics. 2006;6: 3567–3581. doi: 10.1002/pmic.200500853 16705753

[38] Walker J, Vasquez JJ, Gomez MA, Drummelsmith J, Burchmore R, Girard I, et al. Identification of developmentally-regulated proteins in Leishmania panamensis by proteome profiling of promastigotes and axenic amastigotes. Mol Biochem Parasitol. 2006;147: 64–73. doi: 10.1016/j.molbiopara.2006.01.008 16530278

[39] Saxena A, Lahav T, Holland N, Aggarwal G, Anupama A, Huang Y, et al. Analysis of the Leishmania donovani transcriptome reveals an ordered progression of transient and permanent changes in gene expression during differentiation. Mol Biochem Parasitol. 2007;152: 53–65. doi: 10.1016/j.molbiopara.2006.11.011 17204342 PMC1904838

[40] Rochette A, Raymond F, Ubeda JM, Smith M, Messier N, Boisvert S, et al. Genome-wide gene expression profiling analysis of Leishmania major and Leishmania infantum developmental stages reveals substantial differences between the two species. BMC Genomics. 2008;9: 1–26. doi: 10.1186/1471-2164-9-1 18510761 PMC2453527

[41] Depledge DP, Evans KJ, Ivens AC, Aziz N, Maroof A, Kaye PM, et al. Comparative expression profiling of Leishmania: Modulation in gene expression between species and in different host genetic backgrounds. PLoS Negl Trop Dis. 2009;3. doi: 10.1371/journal.pntd.0000476 19582145 PMC2701600

[42] Inbar E, Hughitt VK, Dillon LAL, Ghosh K, El-Sayed NM, Sacks DL. The Transcriptome of Leishmania major Developmental Stages in Their Natural Sand Fly Vector. 2017. Available: http://www.who.int/mediacentre/10.1128/mBio.00029-17 PMC5380837 28377524

[43] Pollack JR. A perspective on DNA microarrays in pathology research and practice. American Journal of Pathology. 2007. doi: 10.2353/ajpath.2007.070342 17600117 PMC1934527

[44] Akopyants NS, Matlib RS, Bukanova EN, Smeds MR, Brownstein BH, Stormo GD, et al. Expression profiling using random genomic DNA microarrays identifies differentially expressed genes associated with three major developmental stages of the protozoan parasite Leishmania major. Mol Biochem Parasitol. 2004;136: 71–86. doi: 10.1016/j.molbiopara.2004.03.002 15138069

[45] Shadab M, Das S, Banerjee A, Sinha R, Asad M, Kamran M, et al. RNA-Seq Revealed Expression of Many Novel Genes Associated With Leishmania donovani Persistence and Clearance in the Host Macrophage. Front Cell Infect Microbiol. 2019;9: 17. doi: 10.3389/fcimb.2019.00017 30805314 PMC6370631

[46] Rastrojo A, Corvo L, Lombraña R, Solana JC, Aguado B, Requena JM. Analysis by RNA-seq of transcriptomic changes elicited by heat shock in Leishmania major. Sci Rep. 2019;9: 1–18. doi: 10.1038/s41598-019-43354-9 31061406 PMC6502937

[47] Ruy PDC, Monteiro-Teles NM, Miserani Magalhães RD, Freitas-Castro F, Dias L, Aquino Defina TP, et al. Comparative transcriptomics in Leishmania braziliensis: disclosing differential gene expression of coding and putative noncoding RNAs across developmental stages. RNA Biol. 2019;16: 639–660. doi: 10.1080/15476286.2019.1574161 30689499 PMC6546399

[48] Christensen SM, Dillon LAL, Carvalho LP, Passos S, Novais FO, Hughitt VK, et al. Meta-transcriptome Profiling of the Human-Leishmania braziliensis Cutaneous Lesion. PLoS Negl Trop Dis. 2016;10: 1–17. doi: 10.1371/journal.pntd.0004992 27631090 PMC5025153

[49] Amorim CF, Novais FO, Nguyen BT, Misic AM, Carvalho LP, Carvalho EM, et al. Variable gene expression and parasite load predict treatment outcome in cutaneous leishmaniasis. Sci Transl Med. 2019;11: 1–10. doi: 10.1126/scitranslmed.aax4204 31748229 PMC7068779

[50] Patino LH, Muskus C, Ramírez JD. Transcriptional responses of Leishmania (Leishmania) amazonensis in the presence of trivalent sodium stibogluconate. Parasites and Vectors. 2019;12: 1–15. doi: 10.1186/s13071-019-3603-8 31300064 PMC6626383

[51] Fiebig M, Kelly S, Gluenz E. Comparative Life Cycle Transcriptomics Revises Leishmania mexicana Genome Annotation and Links a Chromosome Duplication with Parasitism of Vertebrates. PLoS Pathog. 2015;11: 1–28. doi: 10.1371/journal.ppat.1005186 26452044 PMC4599935

[52] Andrade JM, Gonçalves LO, Liarte DB, Lima DA, Guimarães FG, de Melo Resende D, et al. Comparative transcriptomic analysis of antimony resistant and susceptible Leishmania infantum lines. Parasites and Vectors. 2020;13: 1–15. doi: 10.1186/s13071-020-04486-4 33256787 PMC7706067

[53] Farias Amorim C, Novais FO, Nguyen BT, Nascimento MT, Lago J, Lago AS, et al. Localized skin inflammation during cutaneous leishmaniasis drives a chronic, systemic ifn-γ signature. PLoS Negl Trop Dis. 2021;15: 1–19. doi: 10.1371/journal.pntd.0009321 33793565 PMC8043375

[54] Dillon LAL, Suresh R, Okrah K, Corrada Bravo H, Mosser DM, El-Sayed NM. Simultaneous transcriptional profiling of Leishmania major and its murine macrophage host cell reveals insights into host-pathogen interactions. BMC Genomics. 2015;16: 1–15. doi: 10.1186/s12864-015-2237-2 26715493 PMC4696162

[55] Fernandes MC, Dillon LAL, Belew AT, Bravo HC, Mosser DM, El-Sayed NM. Dual transcriptome profiling of Leishmania-infected human macrophages reveals distinct reprogramming signatures. MBio. 2016;7: 1–16. doi: 10.1128/mBio.00027-16 27165796 PMC4959658

[56] Belkaid Y, Mendez S, Lira R, Kadambi N, Milon G, Sacks D. A Natural Model of Leishmania major Infection Reveals a Prolonged “Silent” Phase of Parasite Amplification in the Skin Before the Onset of Lesion Formation and Immunity. J Immunol. 2000;165: 969–977. doi: 10.4049/jimmunol.165.2.969 10878373

[57] Mortazavi A, Williams BA, McCue K, Schaeffer L, Wold B. Mapping and quantifying mammalian transcriptomes by RNA-Seq. Nat Methods. 2008;5: 621–628. doi: 10.1038/nmeth.1226 18516045 PMC13303166

[58] Franzén O, Gan LM, Björkegren JLM. Panglao DB: A web server for exploration of mouse and human single-cell RNA sequencing data. Database. 2019. doi: 10.1007/s00018-013-1491-1 PMC6450036 30951143

[59] Walker DM, Oghumu S, Gupta G, Mcgwire BS, Drew ME, Satoskar AR. Mechanisms of cellular invasion by intracellular parasites Mechanisms of host cell invasion in Leishmania. Cell Mol Life Sci. 2014;71: 1245–1263. doi: 10.1007/s00018-013-1491-1 24221133 PMC4107162

[60] Feijó D, Tibúrcio R, Ampuero M, Brodskyn C, Tavares N. Dendritic cells and leishmania infection: Adding layers of complexity to a complex disease. Journal of Immunology Research. 2016. doi: 10.1155/2016/3967436 26904694 PMC4745329

[61] Kautz-Neu K, Schwonberg K, Fischer MR, Schermann AI, von Stebut E. Dendritic cells in Leishmania major infections: mechanisms of parasite uptake, cell activation and evidence for physiological relevance. Medical Microbiology and Immunology. 2012. doi: 10.1007/s00430-012-0261-2 22983754

[62] Liu D, Kebaier C, Pakpour N, Capul AA, Beverley SM, Scott P, et al. Leishmania major phosphoglycans influence the host early immune response by modulating dendritic cell functions. Infect Immun. 2009. doi: 10.1128/IAI.01447-08 19487470 PMC2715672

[63] Woelbing F, Kostka SL, Moelle K, Belkaid Y, Sunderkoetter C, Verbeek S, et al. Uptake of Leishmania major by dendritic cells is mediated by Fcγ receptors and facilitates acquisition of protective immunity. J Exp Med. 2006. doi: 10.1084/jem.20052288 16418399 PMC2118064

[64] Axelrod O, Klaus S, Frankenburg S. Antigen presentation by epidermal langerhans cells in experimental cutaneous leishmaniasis. Parasite Immunol. 1994. doi: 10.1111/j.1365-3024.1994.tb00315.x 7862465

[65] Liu D, Uzonna JE. The early interaction of Leishmania with macrophages and dendritic cells and its influence on the host immune response. Frontiers in cellular and infection microbiology. 2012. doi: 10.3389/fcimb.2012.00083 22919674 PMC3417671

[66] Chen X, Wu H, Feng J, Li Y, Lv J, Shi W, et al. Transcriptome profiling unveils GAP43 regulates ABC transporters and EIF2 signaling in colorectal cancer cells. BMC Cancer. 2021;21: 1–10. doi: 10.1186/s12885-020-07728-x 33402155 PMC7786480

[67] Toribio R, Ventoso I. Inhibition of host translation by virus infection in vivo. Proc Natl Acad Sci U S A. 2010;107: 9837–9842. doi: 10.1073/pnas.1004110107 20457920 PMC2906859

[68] Shrestha N, Bahnan W, Wiley DJ, Barber G, Fields KA, Schesser K. Eukaryotic Initiation Factor 2 (eIF2) signaling regulates proinflammatory cytokine expression and bacterial invasion. J Biol Chem. 2012. doi: 10.1074/jbc.M112.375915 22761422 PMC3436510

[69] Sacks D, Noben-Trauth N. The immunology of susceptibility and resistance to Leishmania major in mice. Nat Rev Immunol. 2002;2: 845–858. doi: 10.1038/nri933 12415308

[70] Kim BH, Shenoy AR, Kumar P, Das R, Tiwari S, MacMicking JD. A family of IFN-γ-inducible 65-kD GTPases protects against bacterial infection. Science (80-). 2011. doi: 10.1126/science.1201711 21551061

[71] Ngo CC, Man SM. Mechanisms and functions of guanylate-binding proteins and related interferon-inducible GTPases: Roles in intracellular lysis of pathogens. Cellular Microbiology. 2017. doi: 10.1111/cmi.12791 28975702

[72] Yamamoto M, Okuyama M, Ma JS, Kimura T, Kamiyama N, Saiga H, et al. A cluster of interferon-γ-inducible p65 gtpases plays a critical role in host defense against toxoplasma gondii. Immunity. 2012. doi: 10.1016/j.immuni.2012.06.009 22795875

[73] Haldar AK, Nigam U, Yamamoto M, Coers J, Goyal N. Guanylate binding proteins restrict leishmania donovani growth in nonphagocytic cells independent of parasitophorous vacuolar targeting. MBio. 2020. doi: 10.1128/mBio.01464-20 32723921 PMC7387799

[74] Lee SH, Chaves MM, Kamenyeva O, Gazzinelli-Guimaraes PH, Kang B, Pessenda G, et al. M2-like, dermal macrophages are maintained via IL-4/CCL24-mediated cooperative interaction with eosinophils in cutaneous leishmaniasis. Sci Immunol. 2020. doi: 10.1126/sciimmunol.aaz4415 32276966 PMC7385908

[75] Zaph C, Scott P. Th1 Cell-Mediated Resistance to Cutaneous Infection with Leishmania major Is Independent of P- and E-Selectins. J Immunol. 2003;171: 4726–4732. doi: 10.4049/jimmunol.171.9.4726 14568948

[76] Carlsen ED, Liang Y, Shelite TR, Walker DH, Melby PC, Soong L. Permissive and protective roles for neutrophils in leishmaniasis. Clin Exp Immunol. 2015;182: 109–118. doi: 10.1111/cei.12674 26126690 PMC4608500

[77] Ribeiro-Gomes FL, Peters NC, Debrabant A, Sacks DL. Efficient capture of infected neutrophils by dendritic cells in the skin inhibits the early anti-leishmania response. PLoS Pathog. 2012;8. doi: 10.1371/journal.ppat.1002536 22359507 PMC3280984

[78] Bogdan C, Donhauser N, Döring R, Röllinghoff M, Diefenbach A, Rittig MG. Fibroblasts as host cells in latent leishmaniosis. J Exp Med. 2000;191: 2121–2129. doi: 10.1084/jem.191.12.2121 10859337 PMC2193203

[79] Scorza BM, Wacker MA, Messingham K, Kim P, Klingelhutz A, Fairley J, et al. Differential Activation of Human Keratinocytes by Leishmania Species Causing Localized or Disseminated Disease. J Invest Dermatol. 2017;137: 2149–2156. doi: 10.1016/j.jid.2017.05.028 28647347 PMC5786447

[80] Rittig MG, Bogdan C. Leishmania-host-cell interaction: Complexities and alternative views. Parasitol Today. 2000;16: 292–297. doi: 10.1016/s0169-4758(00)01692-6 10858648

[81] Novais FO, Carvalho LP, Passos S, Roos DS, Carvalho EM, Scott P, et al. Genomic Profiling of Human Leishmania braziliensis Lesions Identifies Transcriptional Modules Associated with Cutaneous Immunopathology. J Invest Dermatol. 2015;135: 94–101. doi: 10.1038/jid.2014.305 25036052 PMC4268311

[82] Santambrogio L, Berendam SJ, Engelhard VH. The antigen processing and presentation machinery in lymphatic endothelial cells. Frontiers in Immunology. 2019. doi: 10.3389/fimmu.2019.01033 31134089 PMC6513971

[83] Tamburini BA, Burchill MA, Kedl RM. Antigen capture and archiving by lymphatic endothelial cells following vaccination or viral infection. Nat Commun. 2014. doi: 10.1038/ncomms4989 24905362 PMC4073648

[84] Proud CG. eIF2 and the control of cell physiology. Semin Cell Dev Biol. 2005. doi: 10.1016/j.semcdb.2004.11.004 15659334

[85] Jennings MD, Pavitt GD. EIF5 has GDI activity necessary for translational control by eIF2 phosphorylation. Nature. 2010. doi: 10.1038/nature09003 20485439 PMC2875157

[86] Liu Y, Wang M, Cheng A, Yang Q, Wu Y, Jia R, et al. The role of host eIF2α in viral infection. Virology Journal. 2020. doi: 10.1186/s12985-020-01362-6 32703221 PMC7376328

[87] Jaramillo M, Gomez MA, Larsson O, Shio MT, Topisirovic I, Contreras I, et al. Leishmania repression of host translation through mTOR cleavage is required for parasite survival and infection. Cell Host Microbe. 2011;9: 331–341. doi: 10.1016/j.chom.2011.03.008 21501832

[88] Mahnke A, Meier RJ, Schatz V, Hofmann J, Castiglione K, Schleicher U, et al. Hypoxia in leishmania major skin lesions impairs the NO-dependent leishmanicidal activity of macrophages. J Invest Dermatol. 2014;134: 2339–2346. doi: 10.1038/jid.2014.121 24583949

[89] Schatz V, Strüssmann Y, Mahnke A, Schley G, Waldner M, Ritter U, et al. Myeloid Cell–Derived HIF-1α Promotes Control of Leishmania major. J Immunol. 2016;197: 4034–4041. doi: 10.4049/jimmunol.1601080 27798163 PMC7249608

[90] Hammami A, Abidin BM, Charpentier T, Fabié A, Duguay AP, Heinonen KM, et al. HIF-1α is a key regulator in potentiating suppressor activity and limiting the microbicidal capacity of MDSC-like cells during visceral leishmaniasis. PLoS Pathog. 2017;13. doi: 10.1371/journal.ppat.1006616 28892492 PMC5608422

[91] Hammami A, Charpentier T, Smans M, Stäger S. IRF-5-Mediated Inflammation Limits CD8+ T Cell Expansion by Inducing HIF-1α and Impairing Dendritic Cell Functions during Leishmania Infection. PLoS Pathog. 2015;11: 1–22. doi: 10.1371/journal.ppat.1004938 26046638 PMC4457842

[92] Hammami A, Abidin BM, Heinonen KM, Stäger S. HIF-1α hampers dendritic cell function and Th1 generation during chronic visceral leishmaniasis. Sci Rep. 2018;8: 1–10. doi: 10.1038/s41598-018-21891-z 29472618 PMC5823892

[93] Späth GF, Beverley SM. A lipophosphoglycan-independent method for isolation of infective Leishmania metacyclic promastigotes by density gradient centrifugation. Exp Parasitol. 2001;99: 97–103. doi: 10.1006/expr.2001.4656 11748963

[94] Titus RG, Marchand M, Boon T, Louis JA. A limiting dilution assay for quantifying Leishmania major in tissues of infected mice. Parasite Immunol. 1985. doi: 10.1111/j.1365-3024.1985.tb00098.x 3877902

[95] Ewels P, Magnusson M, Lundin S, Käller M. MultiQC: Summarize analysis results for multiple tools and samples in a single report. Bioinformatics. 2016;32: 3047–3048. doi: 10.1093/bioinformatics/btw354 27312411 PMC5039924

[96] Dobin A, Davis CA, Schlesinger F, Drenkow J, Zaleski C, Jha S, et al. STAR: Ultrafast universal RNA-seq aligner. Bioinformatics. 2013;29: 15–21. doi: 10.1093/bioinformatics/bts635 23104886 PMC3530905

[97] Anders S, Pyl PT, Huber W. HTSeq-A Python framework to work with high-throughput sequencing data. Bioinformatics. 2015;31: 166–169. doi: 10.1093/bioinformatics/btu638 25260700 PMC4287950

[98] Robinson MD, Oshlack A. A scaling normalization method for differntial expression analysis of RNA-seq data. Genome Biol. 2010;11: 1–9. Available: http://genomebiology.com/2010/11/3/R25 10.1186/gb-2010-11-3-r25 PMC2864565 20196867

[99] Liu R, Holik AZ, Su S, Jansz N, Chen K, Leong HS, et al. Why weight? Modelling sample and observational level variability improves power in RNA-seq analyses. Nucleic Acids Res. 2015;43. doi: 10.1093/nar/gkv412 25925576 PMC4551905

[100] Benjamini Y & Hochberg Y. Controlling the false discovery rate: a practical and powerful approach to multiple testing. Journal of the Royal Statistical Society Series B.1995;57: 289–300. doi: 10.2307/2346101

[101] Zheng GXY, Terry JM, Belgrader P, Ryvkin P, Bent ZW, Wilson R, et al. Massively parallel digital transcriptional profiling of single cells. Nat Commun. 2017;8: 1–12. doi: 10.1038/ncomms14049 28091601 PMC5241818

[102] Butler A, Hoffman P, Smibert P, Papalexi E, Satija R. Integrating single-cell transcriptomic data across different conditions, technologies, and species. Nat Biotechnol. 2018;36: 411–420. doi: 10.1038/nbt.4096 29608179 PMC6700744

[103] Stuart T, Butler A, Hoffman P, Hafemeister C, Papalexi E, Mauck WM, et al. Comprehensive Integration of Single-Cell Data. Cell. 2019;177: 1888–1902.e21. doi: 10.1016/j.cell.2019.05.031 31178118 PMC6687398

[104] Macosko EZ, Basu A, Satija R, Namesh J, Shekhar K, Goldman M, et al. Highly parallal genome-wide expression profiling of individual cells using Nanoliter droplets. Cell. 2017;161: 1202–1214. doi: 10.1016/j.cell.2015.05.002 26000488 PMC4481139

[105] McInnes L, Healy J, Melville J. UMAP: Uniform Manifold Approximation and Projection for Dimension Reduction. 2018. Available: http://arxiv.org/abs/1802.03426

[106] Finak G, McDavid A, Yajima M, Deng J, Gersuk V, Shalek AK, et al. MAST: A flexible statistical framework for assessing transcriptional changes and characterizing heterogeneity in single-cell RNA sequencing data. Genome Biol. 2015;16: 1–13. doi: 10.1186/s13059-015-0844-5 26653891 PMC4676162

