# Supplementary figures and images for: In vivo transcriptional analysis of mice infected with Leishmania major unveils cellular heterogeneity and altered transcriptomic profiling at single-cell resolution
Source: PLoS Negl Trop Dis. 2022 Jul 5;16(7):e0010518. doi: 10.1371/journal.pntd.0010518 (PMC9286232; doi:10.1371/journal.pntd.0010518)

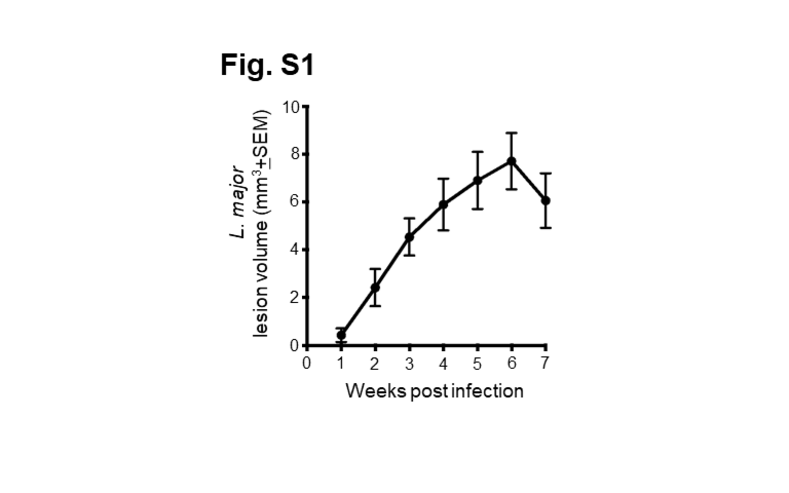

Supplement: S1 Fig — C57BL/6 mice were infected with 2x10^6^ L. major metacyclic promastigote parasites intradermally in the ear and lesion development was monitored over time. Data are pooled from 4 experiments (n = 30) and shown as +SEM. (TIF) [file pntd.0010518.s001.tif]

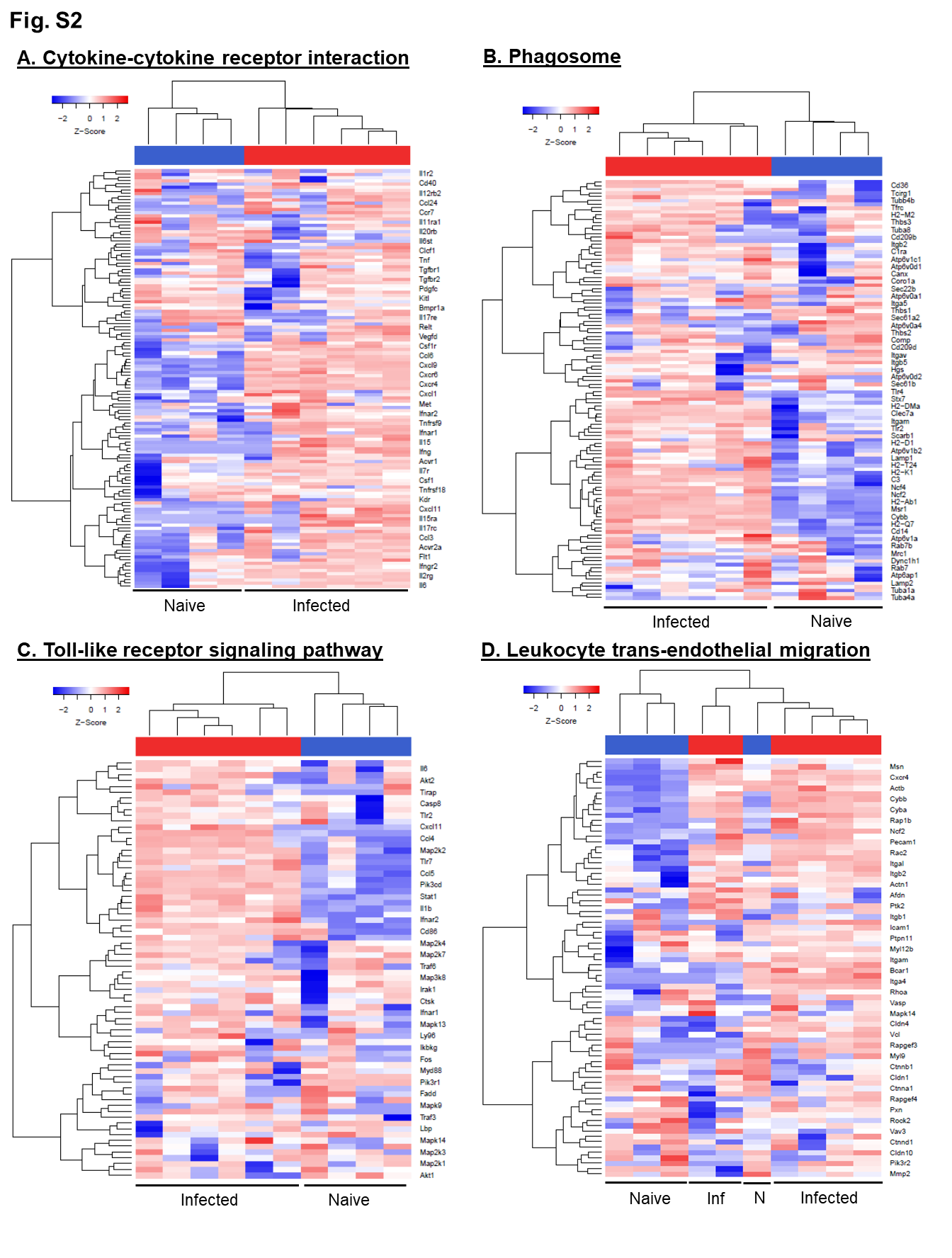

Supplement: S2 Fig — The DEGs involved in the other host immune response pathways by KEGG enrichment analysis (A, B, C and D) in the infected ears compared to naïve mice presented as heat maps. Hierarchical clustering of the expression profile was grouped according to functional categories. Heat maps indicate the FC in L. major infected ear gene expression >2-fold (red) or <2-fold (blue). (TIF) [file pntd.0010518.s002.tif]

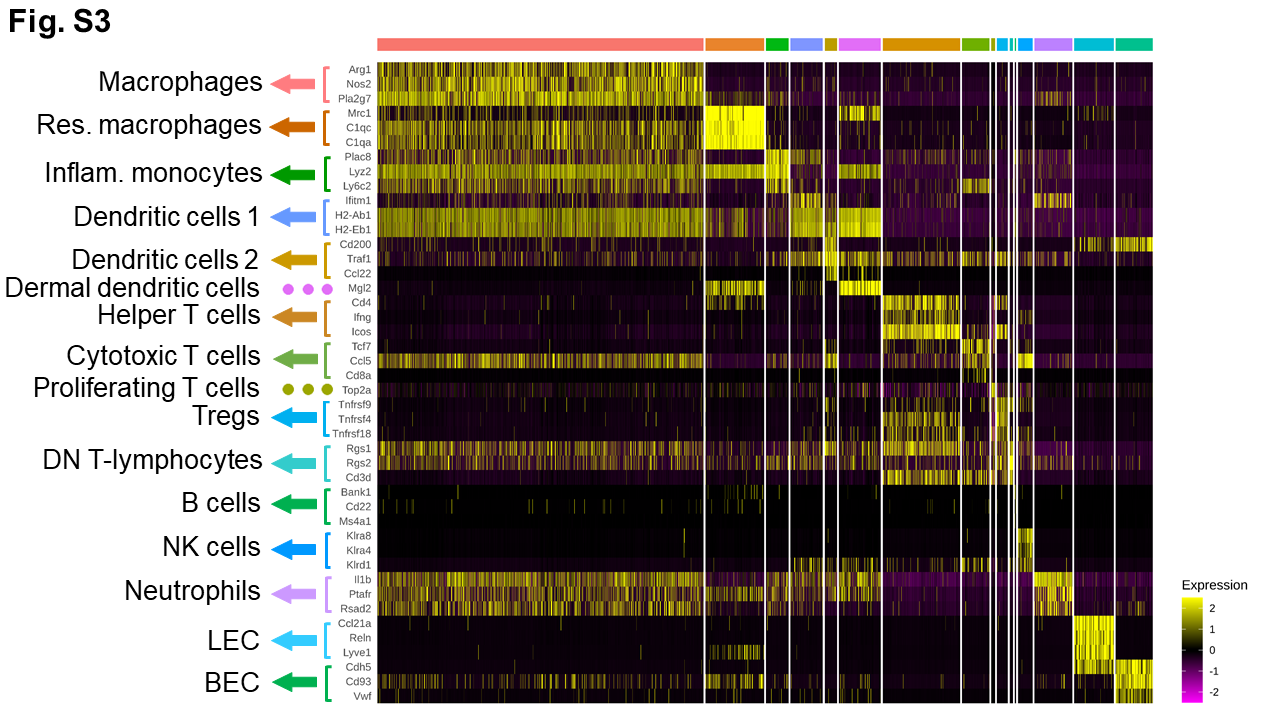

Supplement: S3 Fig — Heat map showing the three highly expressed genes for at least 14 immune cell clusters that were selected along with BECs and LECs. Each column represents a single cell and each row represents an individual gene. Three marker genes per cluster was color-coded and shown on the left. Yellow indicates maximum gene expression and purple indicates no expression in scaled log-normalized unique molecular identifier counts. (TIF) [file pntd.0010518.s003.tif]

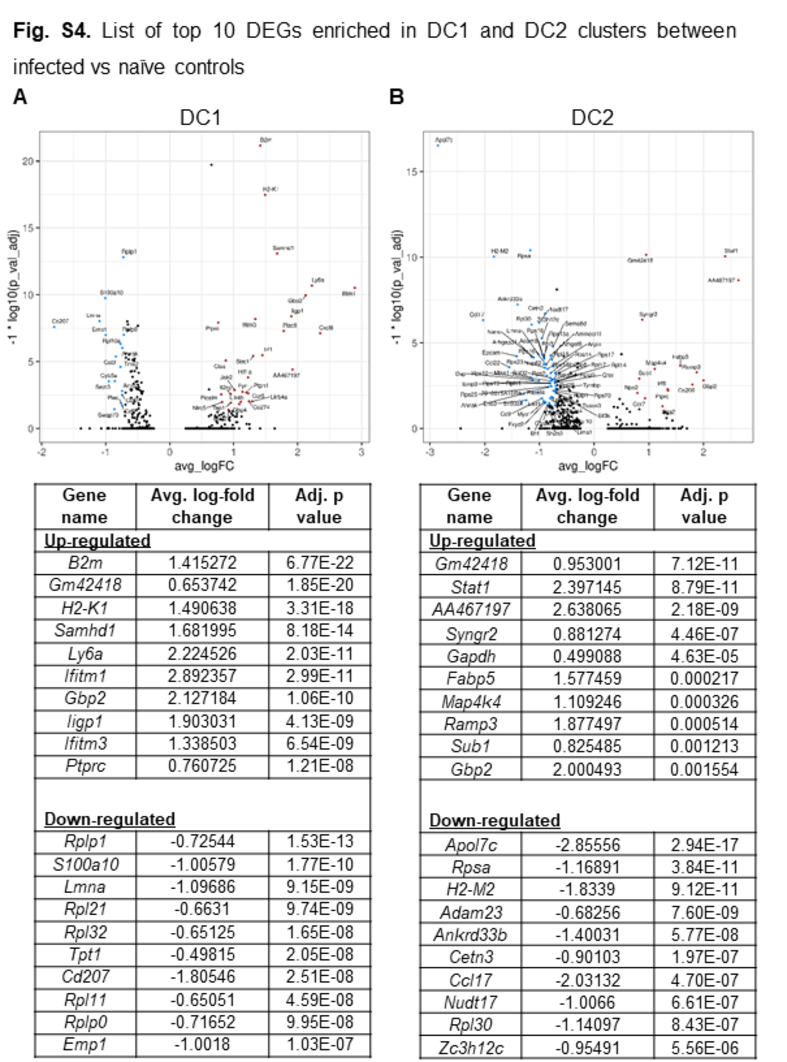

Supplement: S4 Fig — Volcano plot showing the DEGs in dendritic cells (DC1 and DC2) and list includes the top DEGs enriched in DCs following L. major infection. Colored dots indicate genes at least 2 (natural log ~0.693) fold increased (red) or decreased (blue) in infected cells relative to naïve cells with an adjusted p-value < 0.05. (TIF) [file pntd.0010518.s004.tif]

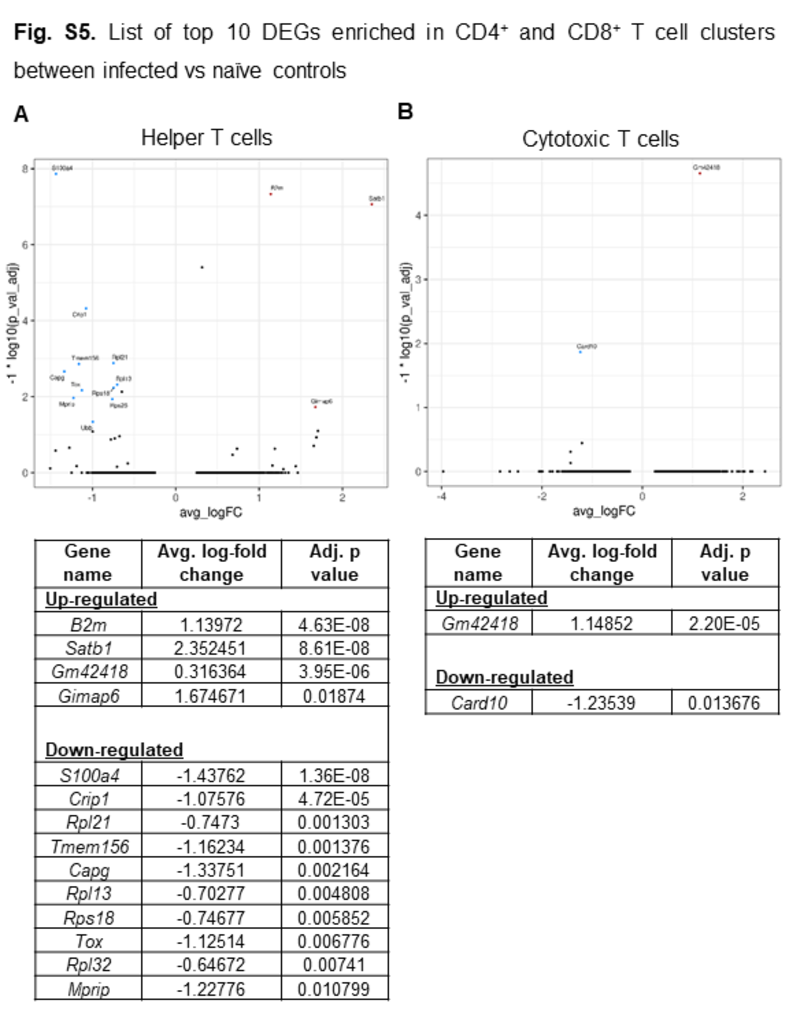

Supplement: S5 Fig — Volcano plot showing the DEGs in CD4+ and CD8+ T cells and list includes the top DEGs enriched in CD4+ and CD8+ T cells following L. major infection. Colored dots indicate genes at least 2 (natural log ~0.693) fold increased (red) or decreased (blue) in infected cells relative to naïve cells with an adjusted p-value < 0.05. (TIF) [file pntd.0010518.s005.tif]

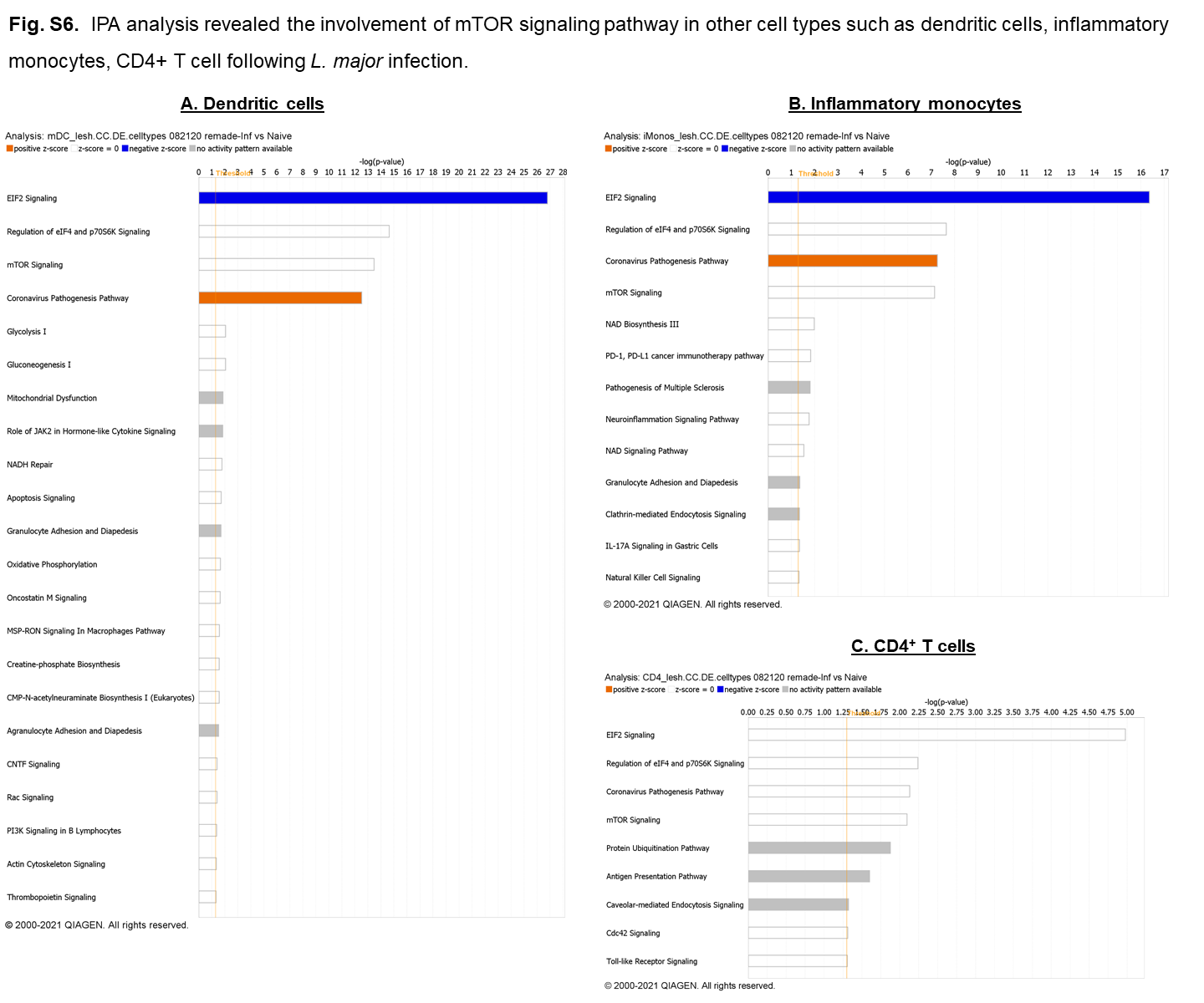

Supplement: S6 Fig — (A-C) Differentially regulated canonical pathways in DCs (A), inflammatory monocytes (B), CD4+ T cells (C) following L. major infection. (TIF) [file pntd.0010518.s006.tif]
