# Supplementary material for: In vivo transcriptional analysis of mice infected with Leishmania major unveils cellular heterogeneity and altered transcriptomic profiling at single-cell resolution
Source: PLoS Negl Trop Dis. 2022 Jul 5;16(7):e0010518. doi: 10.1371/journal.pntd.0010518 (PMC9286232; doi:10.1371/journal.pntd.0010518)
Supplement: S2 Table — (DOCX) [file pntd.0010518.s008.docx]

**Table S2.** DEGs enriched for top 20 KEGG disease pathways.

| **KEGG disease pathway** | **Adj. p value** | **Avg. log-fold.**  **change** | **direction** |
| --- | --- | --- | --- |
| Staphylococcus aureus infection | 1.34E-08 | 6.60 | Up |
| Autoimmune thyroid disease | 3.79E-08 | 6.02 | Up |
| Graft-versus-host disease | 2.19E-07 | 5.43 | Up |
| Allograft rejection | 2.76E-07 | 5.43 | Up |
| Primary immunodeficiency | 7.92E-07 | 8.41 | Up |
| Type I diabetes mellitus | 4.25E-06 | 5.43 | Up |
| Osteoclast differentiation | 3.31E-05 | 8.13 | Up |
| Leishmaniasis | 3.31E-05 | 6.70 | Up |
| Systemic lupus erythematosus | 0.00016 | 5.60 | Up |
| Rheumatoid arthritis | 0.00047 | 6.58 | Up |
| Viral myocarditis | 0.001 | 6.08 | Up |
| Tuberculosis | 0.0023 | 6.13 | Up |
| Asthma | 0.0023 | 2.73 | Up |
| Inflammatory bowel disease (IBD) | 0.0034 | 5.68 | Up |
| Prion diseases | 0.00458 | -0.87 | Down |
| Herpes simplex infection | 0.00472 | 6.19 | Up |
| Measles | 0.00511 | 7.87 | Up |
| Salivary secretion | 0.00703 | 6.63 | Up |
| Pertussis | 0.00703 | 5.29 | Up |
| Toxoplasmosis | 0.00703 | 6.89 | Up |
